# Working with Commercially Available Quantum Dots for Immunofluorescence on Tissue Sections

**DOI:** 10.1371/journal.pone.0163856

**Published:** 2016-09-29

**Authors:** Sandrine Prost, Ria E. B. Kishen, David C. Kluth, Christopher O. C. Bellamy

**Affiliations:** 1 University of Edinburgh, Deanery of Molecular Genetics and Public Health Sciences, Department of Pathology, Queen's Medical Research Institute, 47 Little France Crescent, Edinburgh, EH16 4TJ, United Kingdom; 2 University of Edinburgh, Edinburgh Medical School, Centre for Inflammation Research, Queen's Medical Research Institute, 47 Little France Crescent, Edinburgh, EH16 4TJ, United Kingdom; 3 University of Edinburgh, Deanery of Molecular Genetics and Public Health Sciences, Department of Pathology, Royal Infirmary of Edinburgh, 51 Little France Crescent, Edinburgh, EH16 4SA, United Kingdom; Tohoku University, JAPAN

## Abstract

Quantum dots are semiconductor fluorescent nanocrystals that exhibit excellent characteristics compared with more commonly used organic fluorescent dyes. For many years quantum dot conjugated products have been available in multiple forms for fluorescence imaging of tissue sections under the trademark name Qdot®. They have much increased brightness, narrow emission spectrum, large Stokes shift and photostability compared with conventional organic fluorescent dyes, which together make them the fluorophores of choice for demanding requirements. Vivid Qdots are recent replacements for original Qdots, modified to improve brightness, however this has affected the fluorescence stability in commonly used conditions for immunohistochemistry. We present here our investigation of the stability of original and Vivid Qdots in solution and in immunohistochemistry, highlight the potential pitfalls and propose a protocol for stable and reliable multiplex staining with current commercially available original and Vivid Qdots.

## Introduction

Quantum dots (Q-dots) are semiconductor fluorescent nanocrystals with important advantages as fluorophores compared with commonly used organic fluorescent dyes. Their broad excitation spectra, narrow and symmetrical emission spectra and large Stokes shifts permit more accurate quantification without interchannel bleed through. Q-dots also have excellent fluorescence efficiency: high molar extinction coefficients and quantum yields up to 90% provide a fluorescence output (brightness of emission) many times greater than conventional dyes. In addition, a long excitation state (10-40ns) and photostability about 1000-fold that of conventional organic fluorescent dyes make Q-dots ideally suited for fluorescence image capture and quantification (for reviews [1, 2]).

Taken together, these advantages make Q-dots the fluorophores of choice in demanding fluorescence imaging settings. In particular, for multiplex immunofluorescence staining of tissue sections, they ameliorate the key confounders of low signal/noise ratio and crossover from incompletely filtered overlap between conventional fluorophore emission spectra. Their use with spectral imaging analysis also allows accurate determination of false signal arising from antibody cross-binding.

**Fig 1:**
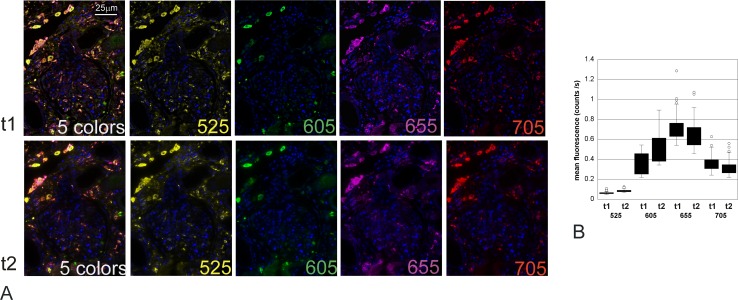
Stability of 5 color immunofluorescence performed with original Qdots: TNFα 525 (yellow), CCR2 605 (green), MHCII 655 (purple), CD68 705 (red) & Qnuclear red (blue), mounted in Qmount and captured 27 months apart. A: unmixed images for each fluorophore and combined 5-colour images at the time of staining (t1) and 27 months later (t2). The cubes were captured (500nm to 720 nm) and unmixed using Nuance. The images shown are false colour images viewed with Clip/Stretch; mapping the lowest 0.01% of the pixels to 0, the highest 0.01% to 255, and linearly interpolating in between. The contrast has been increased 14% in all the photos for more representative printing. No other alterations were performed. The intensity of fluorescence of positive cells for each marker was analysed with the Nuance software and compared between the 2 time points. There was no significant difference in the fluorescence intensity of any marker photographed 27 months apart (p = 0.223, ANOVA). B: Box plot of the mean fluorescence intensity of each fluorophore in the cells of interest (ie thresholded for positivity with the marker labelled by Qdot 525) on the initial image (t1) and after 27 months (t2).

For immunofluorescence, most investigators purchase commercially available Q-dots (tradename Qdot® for InVitrogen and referred as Qdot), which are available in different forms, including as conjugates with antibody, with streptavidin, or in kits to complex Qdots to an antibody of choice (InVitrogen—Molecular Probes). Commercial, 1^st^ generation Qdots (InVitrogen) applied to tissue sections have been found to be stable by ourselves (Qdots 525, 565, 585, 605, 655, and 705 in Qmount medium and others [3]) with no loss of fluorescence over 3 years after initial staining (Fig 1).

From 2012 and beginning with Qdots 625, original Qdots were sequentially replaced with Qdots made using the Vivid technology (Vivid Qdots), although retaining the original name and catalogue number until recently (Table 1). Vivid Qdots had an undisclosed modification of the core/shell manufacture and specific details of the chemical differences of Vivid Qdots from original Qdots have not been released, but they exhibit an improved extinction coefficient [4] (S1 Fig). However, in our hands, Vivid Qdots were less stable than the original Qdots, showing fading, photobleaching and quenching when used according to recommended protocols.

**Table 1:** Dates of reception of Vivid Qdots & lot number.

Qdot	Streptavidin Qdots	First built	Qdot conjugated secondary Ab	First built
**525**	original	na	original	na
**565**	original	na	original	na
**585V**	Dec-13 1344947	Apr-13	Mar-14 1499312	Jan-14 (DαM) Aug-13 (DαR)
**605**	Apr-13 1259352	Jun-12	Mar-14 1567298 & 1537322	Jun-13 (DαM) Oct-12 (DαR)
**625V**	Unknown *	Unknown *	Apr-14 1562999	Unknown *
**655V**	Nov- 12 1144689	Apr-12	Apr-14 1378332	Dec-11 (GαM) Nov-12 (DαG) Nov-12 (DαR) Aug-12 (DαM)
**705V**	Nov-12 1138561	Feb-12	Mar-14 1370933	Aug-12 (GαM) Apr-3 (GαR)

*Date of first use is unknown as initial Qdot625 were not labelled as “Vivid” but were always Vivid technology (InVitrogen personal communication). First build dates were provided by the technical services from Life Technology in March 2016.

DαM: donkey anti-mouse; DαR: donkey anti-rabbit; GαM goat anti-mouse; GαR: goat anti-rabbit

We report here evaluations of the stability and quenching of original and Vivid Qdots in various mounting media, different buffers routinely used in immunochemistry, as well as long term stability. We propose a protocol to maximise stability, and suggest specific controls that allow investigators to confirm the reliability of multiplexing studies performed with the currently available commercial Qdots.

## Material and Methods

### Image capture & analysis

For all comparisons, serial sections were stained with the indicated method and the similar serial fields were imaged at indicated times, and under indicated conditions. All pictures were taken using a spectral camera with tunable filter (Nuance FX; Perkin Elmer) and illumination with a LED at 425nm (Cool-LED PE-2) or 435nm (Cool-LED PE-1000). LED illumination of Qdots was chosen for even, stable illumination without photobleaching of original Qdots [5].

Quantification of the intensity of fluorescence was performed using the Nuance software, with either autothreshold when different slides were compared or selection of region of interest (ROI) through automatic or manual thresholding and use of the same region to analyse the exact same areas when a time course of the fluorescence intensity was performed (slide kept on microscope stage during the whole of the experiment) or using AxioVision (Zeiss) software.

### Immunostaining

#### Preparation of tissue and general information

All stainings were performed on human inflamed liver or kidney tissue. The sections were deparaffinized in xylene (2x 5 min) followed by rehydration through ethanol solutions and 2 minutes wash in tap water. Antigen retrieval was performed in boiling 0.1mM EDTA pH8.0 in a pressure cooker at pressure for 12 minutes.

All antibodies (Ab) and streptavidin Qdots were freshly diluted in Bond diluent (Leica, AR9352) at the appropriate concentrations. A list of the antibodies and concentrations used as well as short hand protocols are available in S1 File.

#### Immunostaining with streptavidin conjugated Qdots (protocol established for the first generation Qdots)

Single staining (short protocol 1 in S1 File): After antigen retrieval, the slides were rinsed with Bond Wash (Leica, AR9590) for 5 minutes, endogenous biotin was blocked using Avidin/Biotin blocking kit (ABBK) as recommended by the manufacturer (Vector SP-2001).

Slides were incubated with the indicated primary antibody for one hour at room temperature, washed in Bond Wash (Leica, AR9590) for 5 minutes and incubated with the appropriate biotinylated secondary antibody (Dako) for 30 minutes at room temperature. After a wash with Bond Wash the slides were incubated for one hour with the streptavidin Qdot. The slides were then counterstained and mounted (see below).

Multiple immunostaining (example of 4 markers) (short protocol 2 in S1 File): After antigen retrieval, the slides were rinsed with Bond Wash for 5 minutes, endogenous biotin blocked (Vector SP-2001) and incubated with serum-free protein block (Dako, X0909) for 10 minutes, prior to simultaneous incubation with primary Ab 1 & 2 (produced in different species) overnight at 4°C. The next day, all remaining steps were performed at room temperature. After a brief wash in Bond Wash the slides were incubated with the first biotinylated secondary antibody diluted in background reducing diluent BKRA (DAKO, S3022) with 1% serum for 30 minutes, washed and incubated with the first streptavidin Qdot. Spare streptavidin and biotin sites were blocked using the ABBK (Vector SP-2001), followed by 10 min incubation in serum-free block (Dako, X0909) and 30 min with the second secondary antibody diluted in BKRA. The slides were then washed and incubated with the second streptavidin Qdot for 30 minutes. A further treatment with serum-free protein block (Dako, X0909) was performed for 10 minutes prior to incubation with primary antibodies 3 & 4 for 1 hour at room temperature. The slides were then washed in Bond Wash; incubated with the third secondary antibody diluted in background reducing diluent BKRA (DAKO, S3022) with 1% serum for 30 minutes, washed and incubated with the third streptavidin Qdot and this was repeated for the 4^th^ primary and secondary Ab. The slides were then counterstained and mounted (see below).

#### Mounting

After incubation with the last streptavidin Qdot the slides were washed in Bond Wash and counterstained with Q-Nuclear Red (LifeTechnologies Q10363) diluted to 1 in 500 in 1x TBS for 5 minutes. After a few seconds rinse in water the slides were dehydrated through 4 ethanol baths, cleared through 2 xylene baths and mounted in Qmount (InVitrogen Q10336- discontinued).

#### Initial multiplex immunostaining with Qdots conjugated secondary antibodies (short protocol 3 in S1 File)

All primary antibodies and streptavidin Qdots were diluted in Bond diluent (Leica AR9352) and secondary antibodies were diluted in BKRA with 1% serum at the appropriate concentration. All washes were performed in Bond Wash pH 7.5 (Leica, AR9590) for 5 minutes unless specified.

The tissues were deparaffinized in xylene, followed by rehydration through ethanol solutions and 2 minutes wash in tap water. Antigen retrieval was performed as described for staining with streptavidin Qdots. Slides were washed with and incubated with serum-free protein block (Dako, X0909) for 10 minutes.

For single staining, slides were incubated with the primary antibody one hour at room temperature. The slides were washed with Bond Wash for 5 min and incubated for 1 hour at room temperature with the appropriate Qdot conjugated secondary antibody. For multiplex staining the first primary Ab was applied overnight at 4°C. After a wash the slides were incubated with the 1^st^ Qdot conjugated secondary antibody for 30 min, washed and the appropriate Fab was used to saturate the primary Ab (10 min). This was followed by a serum free protein block (10 min) prior to incubation with the next primary Ab. The staining steps were repeated for all primary antibodies. Note that for multiplex staining wash time is reduced to 3 minutes and secondary Ab incubation to 30 minutes. Once the staining completed, the slides were washed once more in Bond Wash and counterstained with Q-Nuclear Red (LifeTechnologies Q10363) diluted to 1 in 500 in TBS for 10 minutes rinsed briefly (few seconds) in tap water, dehydrated through increasing concentrations of alcohol, cleared through xylene and mounted in Cytoseal mountant (Richard-Allen Scientific 8310–4) or Ecomount (EM897L Biocare medical).

#### Final method for Qdot conjugated secondaries (and streptavidin Qdot) (short protocol 4 in S1 File)

All primary antibodies and streptavidin Qdots are diluted in BKRA and all secondary antibodies are diluted in BKRA with 1% serum at the appropriate concentration.

All washes are done in BKRA wash (BKRA 1/50 in 50mM Tris) for 3 minutes no more unless specified.

The tissue were deparaffinized, rehydrated and antigens retrieved as described above but washed in TBS pH 8 for 5 minutes, (followed by incubation with Avidin/biotin block and wash if a streptavidin Qdot staining will also be performed) then incubated with serum-free protein block for 10 minutes (Dako, X0909).

The slides were incubated with the first primary antibody overnight at 4 degrees. All following steps were performed at room temperature. The slides were washed and incubated with the first Qdot-conjugated secondary antibody for 35 minutes. After a 3 minutes wash, the slides were incubated with the appropriate FAB (Jackson Immuno, 715-007-003 or 711-007-003) diluted at 1/25 or 1/50 (for αRabbit and αMouse respectively) in BKRA for 10 minutes followed by a serum-free protein block (Dako, X0909) for 2x10 minutes.

The staining steps were repeated for all other markers (up to 6 color staining has been tested) using the appropriate primary antibodies and Qdot-conjugated secondary antibody (Ab) (35 minutes at room temperature for both.

If a streptavidin Qdot labelling was also required (requirement for an amplified signal) the primary Ab incubation was followed by a wash in BKRA 1/50 in 50mM Tris for 3 minutes, incubation with a specific biotinylated secondary antibody diluted in in BKRA with 1% serum for 35 minutes, a further wash and incubation with the chosen streptavidin-conjugated Qdot for 30 minutes.

After a wash the slides were counterstained with Q-Nuclear Red (LifeTechnologies Q10363) diluted to 1 in 500 in BKRA for 5 minutes, rinsed briefly (few seconds) in BKRA 1/50 in 50mM Tris, then dehydrated through increasing alcohol concentrations, cleared through xylene and mounted in Ecomount mountant (Menarini Diagnostics MP-EM897-100) and left at room temperature in the dark overnight to set before imaging.

### Fluorescence stability in solution in various buffers

All assays were done in duplicate in 96 well plates containing 100 μl of each Qdots at the appropriate concentration in the tested buffer (525 & 565: 1/200; 585 &605: 1/1000; 625, 655 & 705: 1/2000). Buffers tested include the following: PBS pH 7.5- Tris 50mM pH 7.5 containing or not 0.1%Tween; 1% PEG, 1%BSA—serum free protein block (Dako X0909)—BKRA (Dako S3022)—TBS- Qdot diluent (2%BSA; 20mM borate buffer pH 8.3 containing 0.05% sodium azide)—20mM borate buffer pH 8.3 containing or not 0.1% Tween. Once BKRA was found to be preventing quenching, we tested various dilutions of BKRA (1/2; 1/5; 1/10, 1/50) in 50mM Tris or in “Bond Wash” containing or not 1% BSA. The excitation of the Qdots was done using illumination at 425 nm and the readings at 525, 565, 585, 615, 655 and 705 nm were done automatically every 30 minutes for 5 to 15 hours.

### Statistical analysis

Data were analysed using Minitab statistical software version 17. Comparisons for the effects of the different factors (including categorical variables such as Qdots, buffers) on fluorescence intensity data were made using Analysis of variance (ANOVA), General Linear Model. Where indicated after fitting a general linear model, multiple comparisons between individual means and factor levels were made with the control group using the Dunnett method with simultaneous 95% confidence intervals and adjusted p values, or pairwise comparisons using the Tukey method and 95% confidence intervals, with adjusted p values. In some experiments a 2-sample t test was used to test for the difference between 2 samples, or, when involving repeated measures of specific cells/microscopic fields, a paired t-test was used to test for differences between the paired observations.

### Tissue sourcing and ethical consent

The clinical tissue samples (inflamed human liver and kidney samples) used in this study were surplus archival paraffin block material originally procured for diagnosis. The archival tissue block samples were fully anonymised to the researchers and as such do not require patient consent. Their use for this study was fully approved by the local clinical governance and ethics committee (Lothian SAHSC and Tissue Governance Unit) with Approval Reference: 07/S1102/21.

## Results

### Stability and quenching of original and Vivid Qdots in various mounting media

Original Qdots protocols from Molecular Probes recommended that stained slides be mounted in PBS or Qmount for viewing. However, after we reported to the company that illumination of Original Qdots mounted in PBS led to quenching visible by eye (not shown), most protocols were updated. Nevertheless, some sites continue to post out-dated protocols that recommend PBS mountant [6].

PBS is still also often recommended as a wash buffer [4, 6–8], but this should avoided whether using original or Vivid Qdots. Original Qdots are extremely stable in their dedicated mounting medium Qmount (Fig 1) when no PBS buffer is used and we established a protocol for 7 color immunofluorescence using Qdots 525, 565 or 585, 605, 625, 655, 705 and Qnuclear red as nuclear counterstain (S1 File).

**Fig 2:**
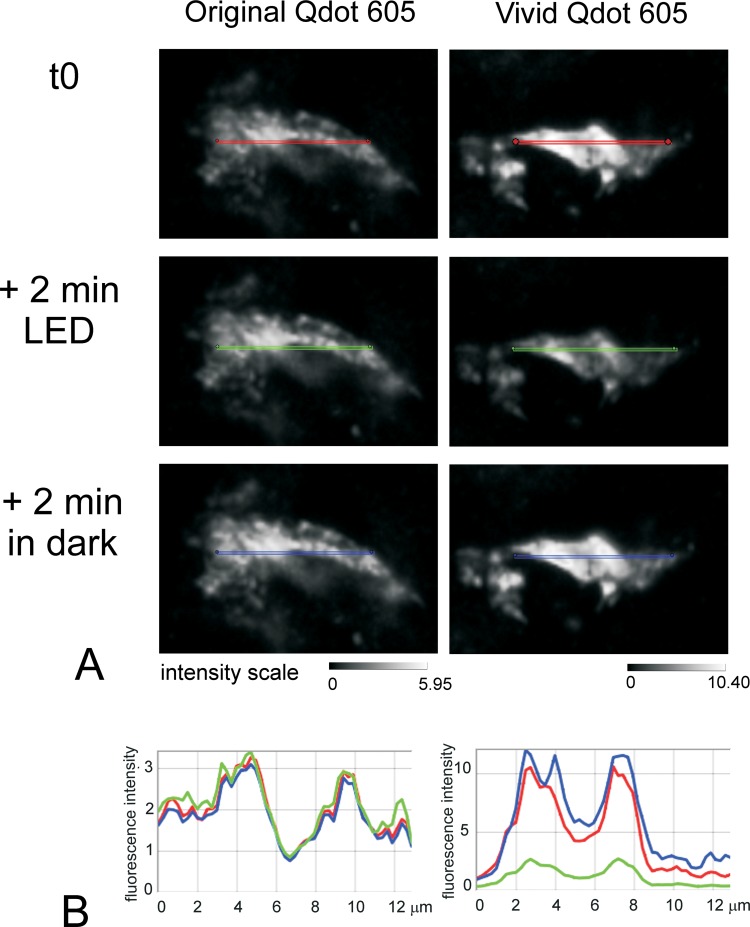
Quenching of Vivid but not original Qdots under LED fluorescence illumination. FFPE serial liver sections were labelled for the marker CD68 using a Labelled StrepAvidin Biotin (LSAB) method with either original streptavidin conjugated Qdot605 or streptavidin conjugated Vivid Qdot605. Fig 2A shows the intensity of fluorescence for one representative cell stained with either the original Qdot (left) or the Vivid Qdot (right): t0 represents the initial capture of the image; the sections were then left under constant illumination with LED 425nm for 2 minutes and finally left for a further 2 minutes in the dark. Note the difference in the intensity scale 0–5.95 for Original Qdot and 0–10.40 for Vivid Qdot. Fig 2B: Intensity profile along the line drawn on each image. Line profiles were drawn on the initial images (t0) using the Nuance software and copied onto the other images. For both original (left) and Vivid Qdot605 (right), the line profile intensities are given scaled to max (red lines: initial intensity; green lines: after 2 minutes illumination with LED 325nm, blue after a further 2 minutes in the dark). The scale is given by the lines which all are 13 μm in length.

**Fig 3:**
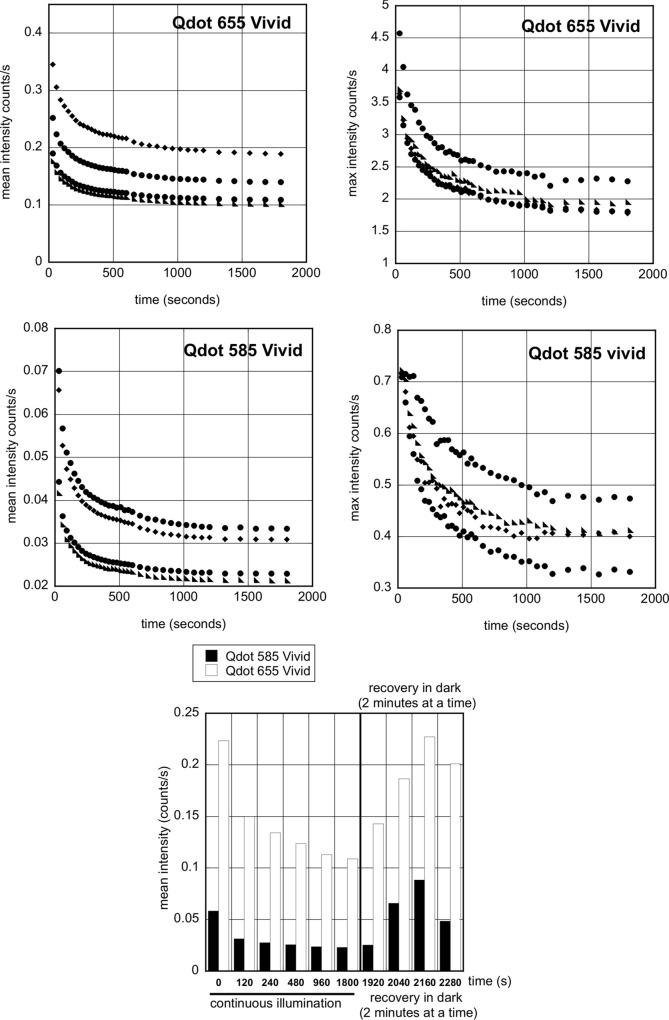
Photoquenching of Qdot 585Vivid and 655Vivid with exposure to LED 435nm. Serial sections of FFPE tissue were stained for CD163 with Qdot 655Vivid (top panel) or Qdot 585Vivid (middle panel) and mounted in Qmount. The stained slides were imaged at the indicated times during 30 minutes under continuous illumination, then after “recovery” in the dark, every 2 minutes. **A:** The graphs show the decrease in mean (left) and maximum (right) fluorescence intensity for Qdot 655Vivid (top panel) and Qdot 585 (middle panel) for four positive cells chosen in the same field of view. **B:** The bottom graph shows the recovery of fluorescence in 2 representative positive cells after the slides are kept in the dark for 2 minutes at a time.

The introduction of new Vivid Qdots in our multiplex protocols led to unexpected results that we eventually traced to the rapid fading of particular Qdots. This was surprising given the excellent and reliable results we had had previously. We compared the intensity of fluorescence of single staining with streptavidin Qdots 525, 565, 585, 605, 655 and 705 from different batches and eventually traced the problem to differences between original and Vivid Qdots. Fig 2 shows an example for Qdot 605: Vivid is about 3-fold brighter than the original Qdot with a commensurate decrease of autoexposure time (91.86 & 46.54ms for original & Vivid Qdot respectively). However continuous illumination for 2 minutes caused quenching of Vivid but not the original Qdots, which reversed after 2 minutes without illumination (Fig 2). Similar photo-quenching was observed for all other Vivid Qdots (examples of Qdot 655 & 585 shown in Fig 3; average 41% and 57% intensity reduction by 300 seconds, respectively, p = 0.008, t test). This instability made Vivid Qdots unsuitable for our quantification studies.

**Fig 4:**
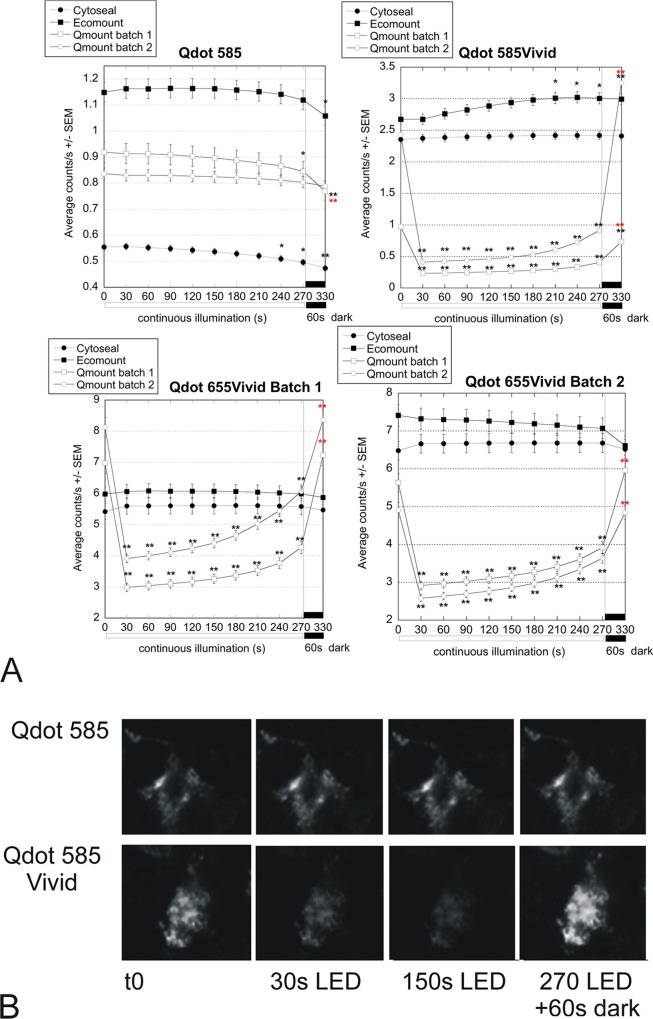
Different stability of Vivid (655 & 585) and original (585) Qdots on immunofluorescence slides mounted in Qmount, Cytoseal and Ecomount. 16 serial sections of human inflamed liver were stained for CD163 with original Qdot (585) or Vivid Qdots (585; 655 batch 1 or 655 batch 2) and imaged at the indicated durations of continuous illumination with LED 435nm (t0 to 270 seconds), then left in the dark for 1 minute before taking the last image. A: Graphs showing the average counts/s +/- SEM of positive regions of interest (ROIs) thresholded on the image of one x40 field captured at t0 and used for analysis of all subsequent images. The fields were matched in the serial sections. Asterisks indicate significant differences (* p<0.05; **p<0.01) compared with the t0 control (black) or the t270 pre-recovery control (red) (ANOVA with Dunnett multiple comparisons) B; representative images of cells labelled with Qdot 585 & Qdot 585Vivid at initial capture (t0), after 30 and 150 seconds illumination (30s LED & 150s LED) and after 270 second illumination followed by 60 second recovery in the dark (270 LED +60s dark)

As Vivid but not original Qdots seemed affected we initially thought the mounting medium could be the issue and compared the then recommended Qmount (2 batches) (inVitrogen/LifeTechnology Q10336) with Cytoseal60 (Thermo Fisher 8310–4) and Ecomount (EM897L Biocare medical). Where still available, the original and Vivid Qdots were compared. Striking differences in stability were found: original Qdots were very stable in Qmount (Fig 4 and also Fig 1, after 27 months), but Vivid Qdots fluorescence decreased sharply with excitation (average quenching in Qmount after 30 seconds illumination was 79% for Qdot 585Vivid (p<0.001; paired t-test), 52% for Qdot 655Vivid (p<0.001), compared with <1% for Qdot 585). There was muted recovery during further constant illumination, and faster recovery when the slides were left in the dark for 1 min (Fig 4). This quenching prevents reliable quantification. Fluorescence in Ecomount or Cytoseal seemed more stable (Fig 4). Following our report to the company, Qmount was discontinued, however, care should be taken with data obtained with Qdots staining in the past few years as people may not have been aware of the issue.

### Long term stability in different mounting media

The fluorescence was more stable in Cytoseal60 and Ecomount than in Qmount (Fig 4). Because of the possibility of using other fluorophores alongside Qdots for greater than 6 colors multiplexing, we tested the stability of Cy3, Cy5 and Alexa dyes 488 & 555 stains mounted in Cytoseal and Ecomount (data not shown). We confirmed that Alexa dyes are stable in Cytoseal [9] as are Cy3 and Cy5. Ecomount is reported to be inappropriate for Alexa(s) dyes (Ecomount product sheet) and this was not tested further.

**Fig 5:**
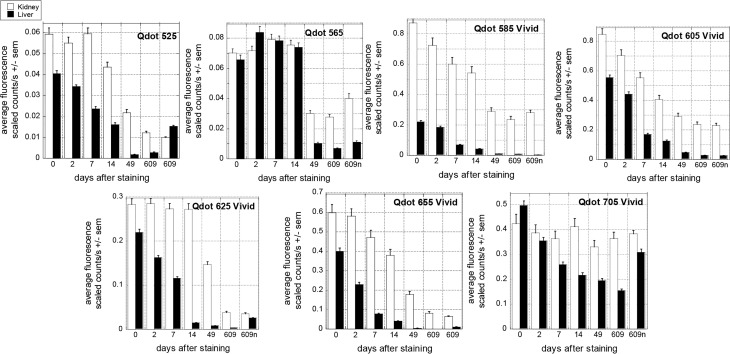
Testing long term fluorescence stability in Cytoseal over 20 months. Serial sections of a block containing both liver and a kidney were stained with the same primary Ab against CD163 and a secondary Ab directly conjugated with the indicated Qdot. The “same” area was imaged at the indicated times after staining and intensity of fluorescence was obtained using Nuance software. A new area was also imaged 609 days after initial staining (609n). The average fluorescence is shown, while Maximum fluorescence in thresholded positive areas, Average and Total areas are available in S2 File together with representative images). Original Qdots (525& 565) were about one tenth as bright as Vivid Qdots but their intensity remained fairly stable in both tissues for at least 14 days. Vivid Qdots photo-bleached rapidly (see text). The difference of fluorescence intensity between liver and kidney sections stained with Qdot 585Vivid is partly due to the choice of the kidney area captured, where the staining was particularly bright.

Although short term fluorescence of all Qdots mounted in Cytoseal or Ecomount then illuminated up to 5 minutes (Fig 4) was stable, we found that Vivid fluorescence was more likely to decrease within a few days after staining (Fig 5 & S2 File), especially in Cytoseal (S2 Fig). Original Qdots in Cytoseal remained fairly stable up to 2 weeks after staining, but not for as long as 49 days, unlike original Qdots in Qmount, which were stable for years (Fig 1). The Vivid Qdots tended to fade more rapidly and to greater degree (Fig 5) (average 28% intensity decrease across all Vivid Qdots in the initial 14 days versus 4% for original Qdots (p<0.001 ANOVA; average 44% decrease across all Vivid Qdots over all intervals to 609 days, compared with 29% decrease for original Q dots; p<0.002). However, there was individual variations: Qdot 705Vivid and 625Vivid were reproducibly more stable than the other Vivid Qdots, especially in kidney sections with the long term fading of Qdot 705Vivid being proportionately not significantly worse than that of original Qdot 565 (p = 0.592, ANOVA). Qdot 585Vivid was the least reliable in our hands.

Intriguingly, the decreased average and maximum fluorescence was greater in the liver than in the kidney sections (Fig 5 & S2 File) despite the mean areas thresholded being fairly constant (S2 File). However, the mean area data should not be over-interpreted, as a minimum of 100 pixels (6.13μm^2^) is imposed during analysis, which might cause underestimation of any reduction of the area thresholded due to decrease in fluorescence (see S2 File).

### Effect of common buffers used in immunostaining on Qdot fluorescence

With this in mind we tested the stability of the Qdots during 6 color multiplex immunostaining.

Six color multiplex immunohistochemistry is typically performed over 2 days with the first primary applied overnight. In these conditions, the first fluorophore is applied 9 to 10 hours before the slide is mounted. We compared the fluorescence of each fluorophore done simultaneously as a single staining and as part of a multi-stain. The results were striking: all single stains were bright, but in multiplex all the Vivid Qdots were quenching during the procedure while original Qdots fluorescence looked stable (data not shown). Therefore in addition to possible quenching during visualisation and photobleaching once mounted, quenching was also occurring during the long staining protocol.

**Fig 6:**
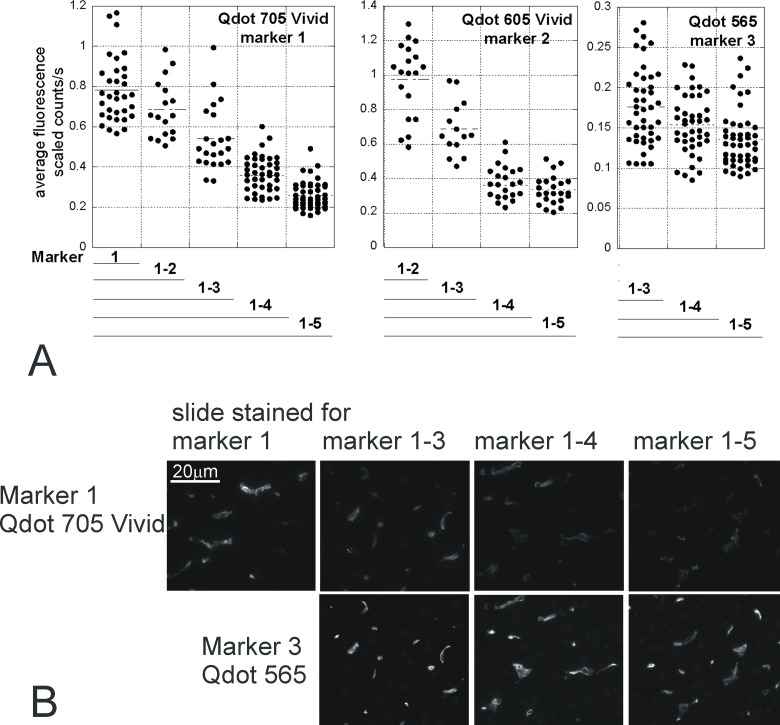
Decreased immunofluorescence during multiplex staining. Serial sections were stained with 1 to 5 different markers (Marker 1, MHCII Qdot 705Vivid; Marker 2, HO1 Qdot 605Vivid; Marker 3, CD163 Qdot 565; Marker 4, CD206 Qdot 655Vivid; Marker 5, CD68 streptavidin Qdot 525). After each staining round, a slide was mounted and imaged. Analysis of the intensity of Qdots 705Vivid, 605Vivid and 565 fluorescence is shown (Qdot 655Vivid & 525 in S3 Fig) for one representative field (aligned in serial sections). Each dot corresponds to one thresholded positive cell. The intensity of fluorescence is affected by the number of staining rounds (p<0.001, ANOVA) and the Qdot type (p<0.001, ANOVA): intensity decreases with the incremental number of subsequent staining rounds, and the reduction is greater for Vivid than original Qdots, (fitted linear regression slopes for absolute and (in brackets) relative brightness change with each additional staining round: -0.02 (-0.11) for Qdot 565; -0.22 (-0.23) for Qdot 605Vivid; -0.14 (-0.17) for Qdot 705Vivid). B: representative images showing the fast decrease of Qdot 705Vivid intensity, and the lesser decrease of Qdot 565 with the number of successive stainings. The Qdot 565 staining on slide 1–5 underwent the same treatments as Qdot 705Vivid on slide 1–3 ie 2 extra rounds of staining. All 705 images are scaled 0–2.2 while 565 images are scaled 0–0.5 (according to the range of fluorescence intensity after initial labelling with each Qdot).

To confirm the quenching was due to the protocol time, we analysed the fluorescence levels of the marker fluorophores (5 markers, 1 counterstain) after each staining round on 6 serial sections stained in parallel (Fig 6). Addition of further multiplex markers (with additional rounds of immunostaining) was associated with incremental reductions in brightness of the index marker, which were pronounced for Qdots 605Vivid and 705Vivid, but minor for the original Qdot 565 (Fig 6 & S3 Fig).

**Fig 7:**
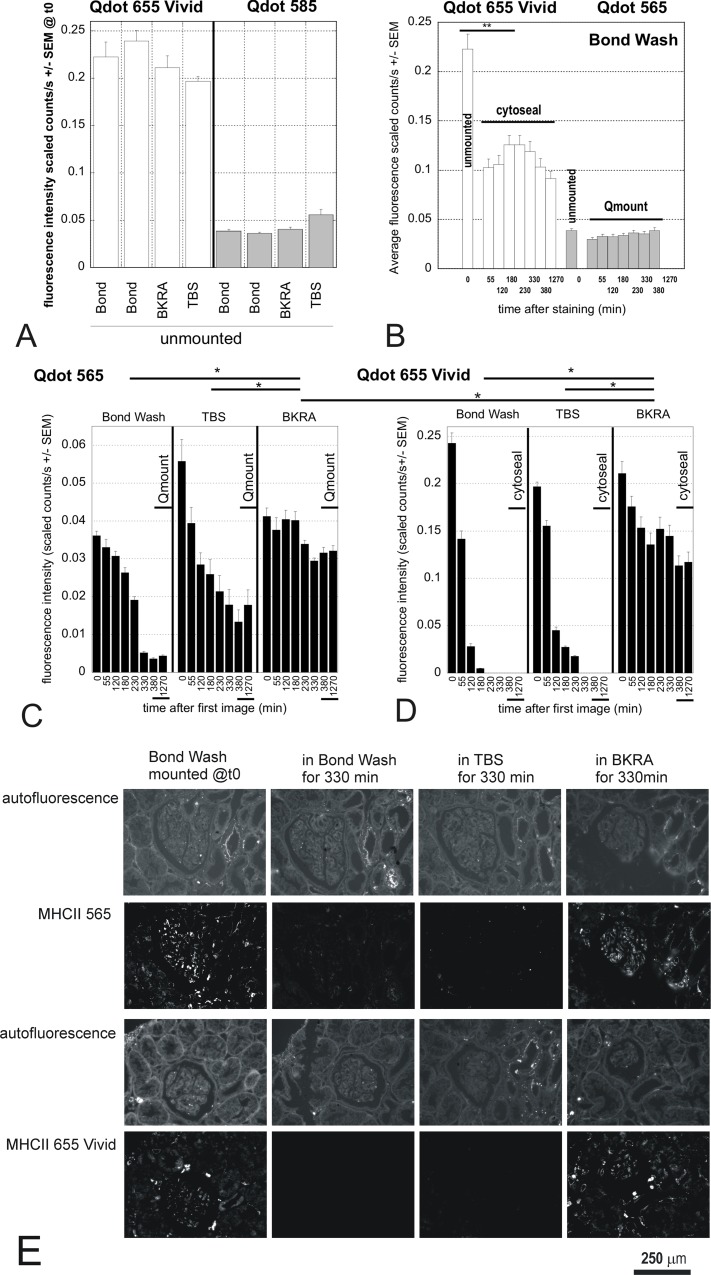
Effect of different buffers and coverslip-mounting agents on fluorescence stability of Vivid and original Qdots. Single MHCII immunofluorescence staining was done on 8 serial sections with either Qdot 565 or Qdot 655Vivid conjugated secondary Ab. After staining, the sections were imaged in the wash buffer used (A). Two control sections were then mounted in cytoseal (Qdot 655Vivid) or Qmount (Qdot 565) and imaged regularly until the next day (B). The remaining slides were left in the indicated wash buffer and imaged regularly for 5 1/2 hours (330 minutes); they were then coverslipped and imaged after mounting (380 minutes), as well as the next day (1270 minutes) (C & D). A: After a single staining round the fluorescence intensity of Qdot 655Vivid and Qdot585 did not vary significantly between the different wash buffers. B: The fluorescence intensity of Qdot 655Vivid mounted in Cytoseal and of Qdot 565 mounted in Qmount after a staining protocol using Bond Wash remained stable in mountant for at least 1270 minutes (p = 0.599, ANOVA), although the fluorescence intensity of Qdot 655Vivid had still reduced after mounting, compared with unmounted slides (** p<0.001, t-test). C &D: Qdot 565 was more stable than Qdot 655Vivid in all buffers, but especially in BKRA (Tukey pairwise comparisons with 95% CI; * p<0.05). D: Qdot 655Vivid signal in Bond Wash and TBS quickly became undetectable due to quenching and photobleaching, but this was significantly improved in BKRA (Bond Wash and TBS grouping separately to BKRA, using Tukey pairwise comparisons with 95% CI; * p<0.05). E: Representative images of the quenching. All images were taken 1270 min after the staining. 1^st^ column: slides were mounted straight after the staining; 2^nd^ to 4^th^ column: slides were mounted after 330 minutes in the indicated buffer. Fluorescence images are shown with the same scale: 0–0.04 for Qdot 565; 0–0.18 for Qdot 655. The system is able to quantify intensity of fluorescence ranging over at least 3 logs; however the requirement to show the fluorescence images with the same scale means for comparison means some images may appear black, even though the quantification was within the compatibility of the system.

We therefore investigated which product in a standard immunofluorescence may affect the fluorescence of the Qdots. Protocol for Qdot staining recommends PBS as wash buffer [4, 7], which we had replaced with “Bond Wash” (Leica, AR9590) as it did not lead to quenching of the original Qdots unlike the recommended PBS. Bond Wash is a proprietary solution that contains ProclinTM950, which we identified with the Invitrogen team as a possible Qdot quencher. We also tested a background reducing agent (BKRA) whose composition is not provided by the manufacturer. We tested the effect of those products as well as TBS as an alternative wash buffer on the intensity of Qdot 565 and Qdot 655Vivid fluorescence (Fig 7). After a single staining round the fluorescence intensity of Qdot 655Vivid and Qdot585 did not vary significantly between the different wash buffers (Fig 7A). Fluorescence after prompt mounting also remained stable for at least 1270 minutes (Fig 7B), although the fluorescence intensity of Qdot 655Vivid had reduced after mounting, compared with unmounted slides (Fig 7B) (p<0.001, t-test). However, when the slides remained incubating in the buffers at room temperature (for up to 330 minutes; Fig 7C) before mounting, there was an appreciable quenching and photobleaching that varied according to both the Qdot and buffer (Fig 7C & 7D)(p<0.001 ANOVA). Qdot 565 was more stable than Qdot 655Vivid in all buffers, and Qdot 655Vivid in Bond Wash or TBS soon became almost undetectable due to quenching and photobleaching (Fig 7D). However, both the Vivid and original Qdots in BKRA were significantly better protected from the loss of fluorescence compared with Bond Wash or TBS (p<0.001, ANOVA with Tukey pairwise comparisons).

Six months after mounting, the fluorescence intensity of Qdot 565 left in BKRA for 1270 minutes was still similar to the original fluorescence level (0.033 +/- 0.001 counts/s for original area at t0 vs 0.037 +/- 0.001 in both new area and original areas 6 months later). Qdot 565 left in Bond Wash lost about half its intensity (0.036 +/-0.001 counts/s vs 0.018 +/- 0.000), while that left in TBS showed no recognisable positive cells (only a few positive pixels remained). For Qdot 655Vivid, fluorescence was virtually undetectable when incubated in either Bond Wash or TBS (only sparse positive pixels, but no identifiable positive cells). In the BKRA-incubated slides, the Vivid fluorescence was reduced to about one fifth in fields not previously imaged (0.117 +/-0.005 to 0.022 +/-0.003 scaled counts/s). In areas previously imaged, the fluorescence had gone almost completely, with no recognisable positive cells, so quantification was not performed.

Taken together, these data show that significant photobleaching and quenching occur both during the staining process and after mounting, particularly for Vivid Qdots, and that different buffers affect the susceptibility of Qdots to these instabilities.

**Fig 8:**
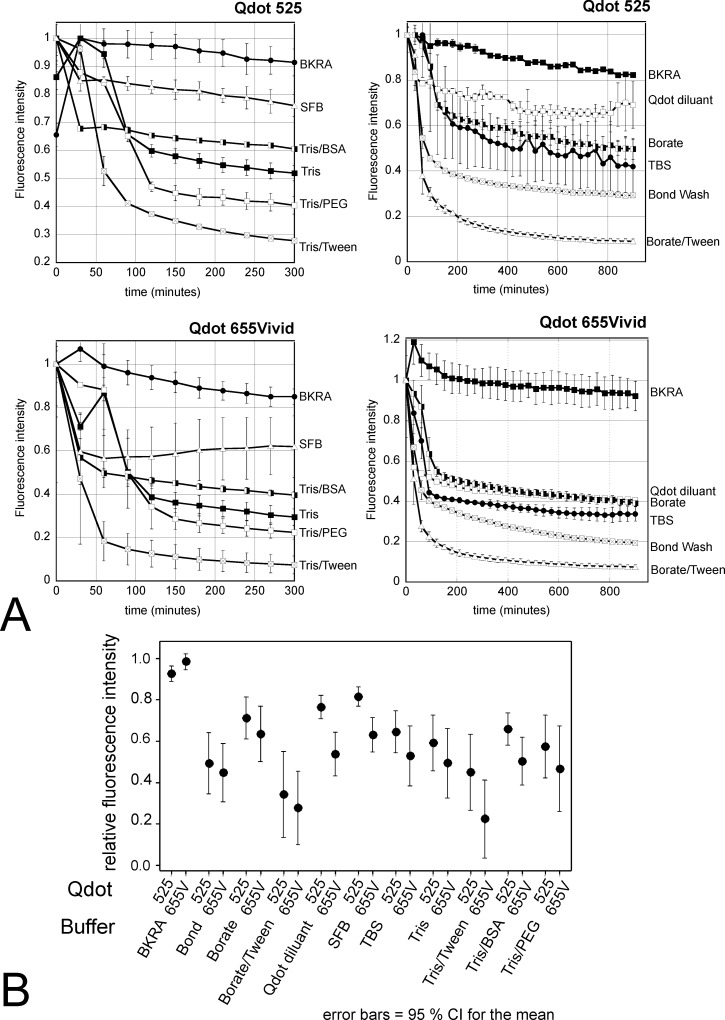
Example of stability of Qdots in different buffers in solution. A: Example of Qdot 565 & Qdot 655Vivid. Stability of all the Qdots were tested in 96 well plates in the buffers and solutions used in our multiplex staining, as well as other common buffers. Two dilutions of each Qdot were analysed in duplicate. B: The interval plot summarises (95%CI) the relative intensity (with 95% confidence limits) for Qdot 565 and Qdot 655Vivid in the different buffers, showing that BKRA (DAKO background reducing agent) provides the best stability. Intensity in all other buffers became significantly reduced for at least one Qdot within 30–90 minutes (ANOVA with Dunnett comparison with t0 as control). Addition of Tween to either Tris or Borate buffer further decreased fluorescence stability for all Qdots. All Qdots tested behaved similarly in solution (data not shown). SFB: serum free block.

Given that our protocol respected pH and other conditions recommended [8, 10], we tested the stability of all Qdots with time in each individual diluent and wash solution (Fig 8) using a fluorimeter. We tested all Qdots in solution in Bond, PBS (recommended as wash buffer in Qdots immunostaining protocols), TBS, BKRA, borate buffer, Qdot diluent, borate & tween buffer, serum free protein block, Tris, Tris tween, Tris PEG, Tris BSA for up to 1500 minutes. We found that Qdots in solution faded progressively with time, to a variable degree that depended on the diluent buffer (example plots for Qdot 525 & 655Vivid in Fig 8). All Qdots were most stable in a proprietary background reducing agent, BKRA (Dako), which preserved fluorescence intensity significantly better than any other buffer (adjusted p<0.001; ANOVA with Tukey pairwise comparisons) with a mean relative intensity during the first 300 minutes of 0.95 of the time zero value, compared with 0.72 for the next best buffer (SFB) and 0.33 or less for buffers containing Tween (Fig 8). Indeed, Qdot 565 or Qdot 655Vivid in BKRA showed no significant change in brightness with time (p = 0.157 and p = 0.586 respectively, ANOVA). We were unable to obtain the exact composition of this buffer, but knowing that Qdots are shipped in Borate buffer and that BKRA is a borate-based buffer at pH 8.35 we tested other borate buffers, without reaching the stability provided by the BKRA. We repeated the test multiple times and confirmed that all Qdots, whether original or Vivid, are most stable in BKRA or dilutions of BKRA in TRIS (Fig 8).

### Establishment of a reliable protocol for multiplexing with Qdots

**Fig 9:**
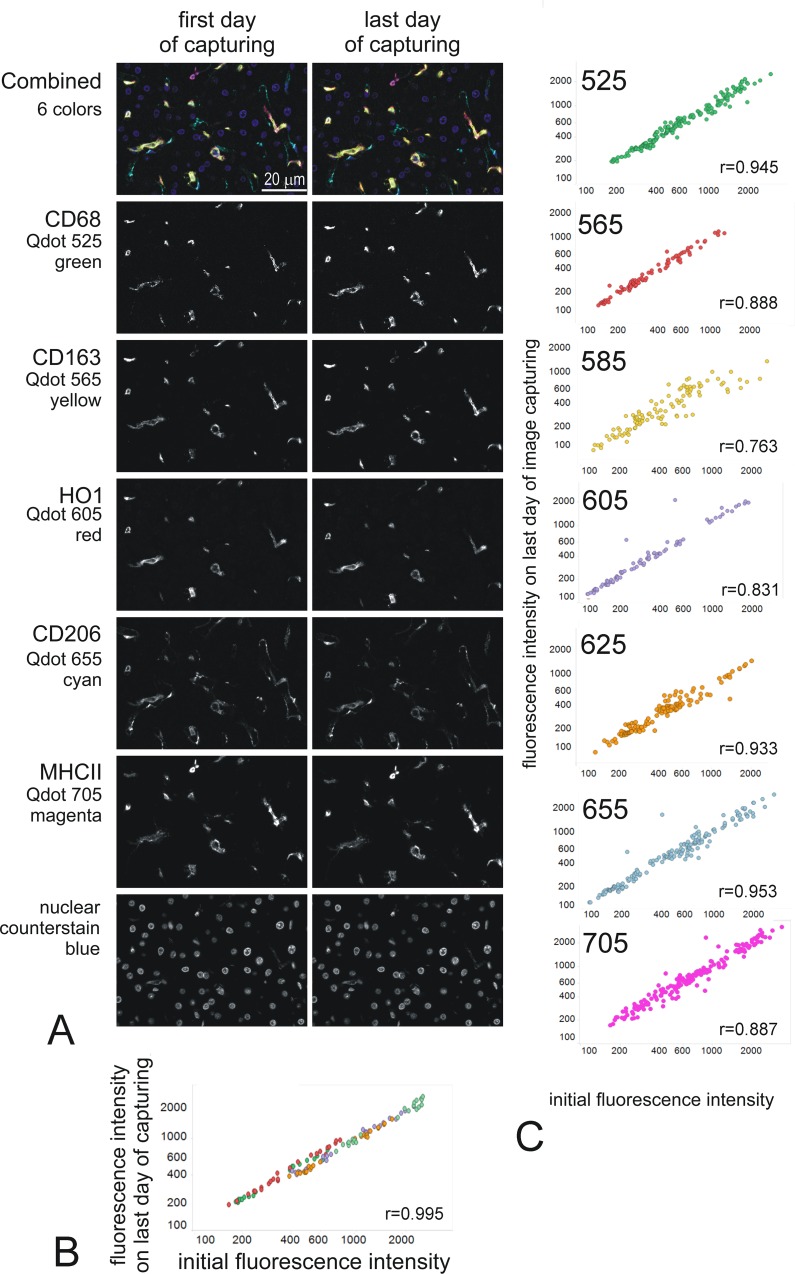
Reproducible stability of Qdot fluorescence in multiplex immunofluorescence. A: representative images (about 1/8 of the actual captured images) of a 7 colors staining taken on the day of staining and on the last day of image capturing of the experiment (day 4 for the example shown). The markers and Qdots used are indicated on the left of the images. The fields were matched as closely as possible. The images shown are intensity images spectrally unmixed with Nuance, at the same scale on first and last day of capturing. The top 2 images are combined images for all 6 makers, with false color as indicated under each marker. B: plot showing the intensity of fluorescence on the initial (x-axis) versus the last (y-axis) day of capturing (day 4) (16 x20 fields; intensity in counts/s). The linearity confirms the stability of the staining, (Pearson coefficient r = 0.095 p<0.001). C: Stability plot for the indicated Qdots used in 11 6-colour multiplex experiments. Data is expressed as mean fluorescence intensities (counts/s) per microscope field, analysed initially (D0) and various days later. All data were analysed by the same operator, using AxioVision and manual thresholding for a cell-specific marker to define the cells of interest. The plots show correlations between the initial intensity and the final intensity for 6 Qdots pooled from 11 different staining batches. The specific fluorescence intensity did not change significantly with time (daily to 5 days after initial staining) compared with the initial measured intensity for all Q dots taken together (Pearson coefficient “r” is indicated for each Qdot all with p<0.001).

A new protocol (S1 File) for multiplexing was tested using BKRA diluted 1/50 in TRIS for all washes and mounting in Ecomount. This gave reliable results with stable fluorescence. This protocol has now been used multiple times with 6-colour multiplex experiments using a combination of 5 Qdots and Qnuclear red as nuclear counterstain, with reproducibly stable results (Fig 9). In all multiplex staining, we now include tests for the stability of each Qdot over the sequential staining steps, for the stability of each label with time in mountant, as well as the usual controls for the antibody staining itself (Table 2).

**Table 2:** Example of controls for a 5-marker multiplex immunofluorescence. Eleven serial sections of a control block are stained with the experimental samples and serve as controls.

1	2	3	4	5	6	7	8	9	10	11
xxxxx	xxxxx	xxxxx	xxxxx	xxxxx	xxxxx	xxxxx	xxxxx	xxxxx	xxxxx	xxxxx
Marker 1		Marker 1	Marker 1	Marker 1	Marker 1	Marker 1	Marker 1	Marker 1	Marker 1	Marker 1
Goat αMouse	GoatαMouse	Goat αMouse	Goat αMouse	Goat αMouse	Goat αMouse	Goat αMouse	Goat αMouse	Goat αMouse	Goat αMouse	Goat αMouse
Marker 2	Marker 2		Marker 2	Marker 2	Marker 2		Marker 2	Marker 2	Marker 2	Marker 2
Donkey αRabbit	Donkey αRabbit	Donkey αRabbit	Donkey αRabbit	Donkey αRabbit	Donkey αRabbit		Donkey αRabbit	Donkey αRabbit	Donkey αRabbit	Donkey αRabbit
Marker 3	Marker 3	Marker 3		Marker 3	Marker 3			Marker 3	Marker 3	Marker 3
Donkey αMouse	Donkey αMouse	Donkey αMouse	Donkey αMouse	Donkey αMouse	Donkey αMouse			Donkey αMouse	Donkey αMouse	Donkey αMouse
Marker 4	Marker 4	Marker 4	Marker 4		Marker 4				Marker 4	Marker 4
Donkey αRabbit	Donkey αRabbit	Donkey αRabbit	Donkey αRabbit	Donkey αRabbit	Donkey αRabbit				Donkey αRabbit	Donkey αRabbit
Marker 5	Marker 5	Marker 5	Marker 5	Marker 5						Marker 5
αMouse IgG3biot	αMouse IgG3biot	αMouse IgG3biot	αMouse IgG3biot	αMouse IgG3biot	αMouse IgG3biot					αMouse IgG3biot
**5 markers Qred**	**No primary control**	**No primary control**	**No primary control**	**No primary control**	**No primary control**	**1 marker no Qred**	**2 markers no Qred**	**3 markers no Qred**	**4 markers no Qred**	**5 markers no Qred**
**stability**										
**Controls for crossbinding**						**Controls for the loss of fluorescence during the full staining process**				

For stability test, on day zero, 20 (x20 objective) to 40 fields (x40 objective) of slide 1 are imaged using Nuance. At regular intervals (at least once a day for the time of the capturing), 4 fields are re-captured and the intensity of fluorescence for each marker is compared with day zero. The fields chosen are changed every day to avoid potential confounding from photobleaching (which does not happen in our current experimental conditions). Comparisons of slides 1 to 6 are used for cross binding control and 7 to 11 for quenching during the staining protocol.

## Discussion

The present data show that recent alterations of the Qdots chemical structure to improve their brightness have rendered the Qdots fluorescence more sensitive to buffers and mounting agents. Although the Vivid Qdots are much brighter than their original counterparts, we show here that both photostability and long term stability are reduced in some conditions relevant to immunofluorescence staining of tissue sections, including the initially recommended mounting medium in which photobleaching was observed. Fading is easily overlooked, for example, in experiments using auto-thresholding, it could manifest as smaller areas selected leading to an apparent unchanged mean of fluorescence. To prevent this pitfall, review of other parameters (max & mean fluorescence) is recommended. Nevertheless, we have defined here conditions and controls for reliable multiplex immunofluorescence with Qdots whereby photostability is maintained, allowing their considerable advantages over other fluorophores to be realised.

Different types of quantum dots are classified according to their composition (for basic information [11], for detailed information see [12]). The Qdots used in the present study are described as core-shell Qdots, with a core of CdSe or CdTe, encapsulated in a shell of ZnS further coated with a polymer and either streptavidin or Ab [13]. Coating quantum dots with shells improves quantum yield through passivation [14] and can affect other photo-physical properties [14–18] and many studies show how alteration of the surface coating alter various characteristics such as solubility or toxicity (for reviews see [2, 19]).

Vivid Qdots have higher extinction coefficient and quantum yield (S1 Fig & LifeTechnology, personal communication) leading to much increased brightness compared with the original Qdots. This was achieved through “modification of the core/shell manufacture” with “structural and compositional improvements to the semiconductor nanocrystal “while “the polymer coating(s) remained unchanged” (LifeTechnology, personal communication). However the photostability of the Quantum dot is also related to the shell. Grabolle *et al* [17] described how a thicker shell increases the stability of the Quantum dot but at the cost of imperfections that reduce the radiation-less recombination and thus the quantum yield. They suggested that a reliable shell can only provide a fluorescence quantum yield of about 50% and that it is important to find a compromise between adequate particle stability and high quantum yield. They described how some Quantum dots they tested had high quantum yield but low stability and called for a standardised quality control test for Quantum dots [17].

The present data show that Qdot 585Vivid is less stable than other Vivid Qdots, while Qdots 705Vivid and 625Vivid are the most stable of the Vivid range in experimental conditions relevant to tissue section immunofluorescence. Without detailed information about the chemistry of the Qdots we used it is difficult to speculate on the reasons for these differences, but it is reassuring that we were able to find a protocol in which the fluorescence of all Vivid Qdots was stable enough for reliable quantification of multiplex immunohistochemistry. Original Qdots remain the most stable of the Qdots we have worked with, and we believe that it is possible to obtain those as a custom order, although the excellent matched mounting media that was initially recommended is now unavailable.

There is a constant drive to optimise products and this is particularly true for Q-dots, which exhibit excellent properties and have great potential in diverse applications [12, 20–24]. With such diversity, vendors cannot pre-test all relevant user settings and this was highlighted by the challenges we faced after the replacement of Qdots with Vivid Qdots. While all Qdots were apparently stable under the vendor’s testing conditions, and also in our hands in short term experiments (single staining), more challenging experimental conditions highlighted the inadequacy of the mounting media initially recommended, as well as the destabilising effect on the fluorescence of various commonly used buffers (Table 3). Further alterations in Q-dot synthesis are likely with developments in chemistry or production of the Q-dots [18, 22, 23, 25, 26] and these will not necessarily be flagged to customers, as was the case for the Vivid technology. Until a reliable and recognised quality standard is set that provides customers with confidence about the reliability and reproducibility of the product in their application, researchers must ensure that the parameters important to their study are checked and controlled. The risk is greatest when researchers lacking experience in a particular area such as tissue histochemistry or fluorescence imaging, turn to it for a particular focused application, relying on a “kit” approach to the product, rather than more fundamental experience and controls to validate their intended use.

**Table 3:** tested and recommended products for working with Qdots.

	Original	Vivid	Notes
**PBS**	Quenching	Quenching	**avoid** (even if manufacturer’s protocol still recommends all solutions in PBS)
**TBS**	photobleaching	Quenching & photobleaching	**avoid**
**Bond Wash**	photobleaching	Quenching & photobleaching	**avoid**
**Tween @ 0.1%**	quenching	quenching	“stable up to 0.5% “in manufacturer information
			**avoid**
**BSA**	Provides some protection from quenching (in Tris)		Test further if needed
**Qdot diluant**	Good—Moderate quenching in solution		Used in multiplex
**Borate buffer**	Moderate quenching in solution		Use with caution
**TRIS**	quenching		Use with caution to dilute BKRA if cost needs to be kept low
**BKRA**	stable		Used in multiplex
**SFB**	Fairly stable		Used in multiplex
**Qdots**			
**525**	Stable		Original Qdot–very stable and reliable in most buffer tested
**565**	Stable		Original Qdot–very stable and reliable in most buffer tested
**585V**	Least stable in all buffer tested including BKRA		Avoid if possible Control for stability at all times
**605V**	Average stability		Control for stability at all times
**625V**	Good stability		Control for stability
**655V**	Average stability		Control for stability at all times
**705V**	Good stability overall		Control for stability
**Cytoseal**	Good stability for 1 to 2 weeks		Control for loss of fluorescence with time if quantitative analysis over more than 1 day
**Ecomount**	Good stability for at least 2 weeks		recommended

Fluorescence markers are a mainstream tool in biological research and advances in detection and analysis technologies allow the detection of multiple targets in individual cells. The complexity of multiplex immunofluorescence requires an optimised set-up that includes the hardware, the imaging software and the assays to achieve and confirm specificity, sensitivity and most importantly reproducibility. Even semi-quantitative evaluation requires optimisation and control of all the parameters in the staining and imaging chain, including stability of the staining. The choice of fluorophore is crucial and many dyes with different characteristics and qualities are available and developed regularly [27, 28]. Four key characteristics of a suitable fluorophore can be enumerated as: 1. Bright, so that low level target can be detected; this also reduces the capture time and risk of photobleaching, so high extinction coefficient and quantum yield are an advantage. 2. Narrow emission spectrum with a large Stokes shift; this allows a better and specific detection of the emission, without spectral overlap between fluorophores (which filters only incompletely correct) and bleed through (cross-bleeding). 3. Excellent photostability, as quantification cannot be achieved with a dye that photobleaches significantly under exposure. 4. No quenching from the local environment. Within commercially available dyes, few, if any can match the performance of Qdots in terms of these criteria, including extinction coefficient 10- to 500-fold greater and high quantum yields, with a fluorescence life time 10–20 times longer than other organic fluorophores (30-100ns versus <5ns), together generating a high signal to noise ratio [29]. The Qdot series spans the capture spectrum (emission peaks between 525 and 800nm) and has the advantage of a broad excitation, similar for all Qdots, while large Stokes shifts and narrow symmetrical emission spectra eliminate spectral overlap between absorption and emission. Their brightness allows labelling of low level target without large non-linear amplification steps, cross-bleeding is minimal, and stability in the present protocol is superior to many other fluorophores (Alexa dyes, FITC, TRITC are far less photostable in our hands, although Cy3, Cy5 are photostable [5]. These features together maximise detection of fluorescence without interference, which makes quantification more reliable. While still rather little used compared with less bright and apparently less stable fluorophores such as Alexa dyes [30], Qdots with the present protocol are the fluorophore of choice for multiplex semi-quantitative or quantitative study.

## Conclusion

In conclusion, with the present suggested protocol to maintain stability, we highly recommend the use of Qdot fluorophores for multiplex immunofluorescence staining of tissue sections. We also recommend that the photostability—whether quenching or bleaching—of each fluorophore is controlled for during the staining process and after coverslipping until after image capture. These validation data should be available with publication. We suggest some caution when interpreting data published without such controls, particularly with newer purchased Qdots evaluated under the commonly used conditions shown here to promote instability, of which experimenters would likely have been unaware and not tested.

## Supporting Information

S1 Fig Information on Original & Vivid Qdots. (PDF)

S2 Fig Comparison of Qdot stability in Cytoseal & Ecomount after 14 days. (PDF)

S3 Fig Supplementary graph for Fig 6 showing the additional marker 4 and marker 5 fluorescence intensity levels. (PDF)

S1 File Protocols. (PDF)

S2 File Supplementary data to Fig 5 & representative pictures showing intensity comparison of the same field of view taken at various times. (PDF)

S3 File crude data for all figures. (XLSX)
