# Supplementary material for: Working with Commercially Available Quantum Dots for Immunofluorescence on Tissue Sections
Source: PLoS One. 2016 Sep 29;11(9):e0163856. doi: 10.1371/journal.pone.0163856 (PMC5042461; doi:10.1371/journal.pone.0163856)
Supplement: S1 Fig — (PDF) [file pone.0163856.s001.pdf]

Product	350 nm	405 nm	488 nm	532 nm
Qdot® 525	710,000	360,000	130,000	N/A
Qdot® 565	1,900,000	1,100,000	290,000	140,000
Qdot 585	3,500,000	2,200,000	530,000	305,000
**Qdot® VIVID® 585**	**7,600,000**	**5,300,000**	**1,400,000**	**240,000**
Qdot 605	4,400,000	2,800,000	1,100,000	580,000
**Qdot® VIVID® 605**	**11,300,000**	**7,700,000**	**1,500,000**	**420,000**
Qdot 625	14,700,000	9,900,000	2,700,000	870,000
**Qdot® VIVID® 625**	**14,700,000**	**9,900,000**	**2,700,000**	**870,000**
Qdot 655	9,100,000	5,700,000	2,900,000	2,400,000
**Qdot® VIVID® 655**	**16,000,000**	**11,000,000**	**3,600,000**	**2,200,000**
Qdot 705	12,900,000	8,300,000	3,000,000	2,100,000
**Qdot® VIVID® 705**	**14,000,000**	**10,000,000**	**3,400,000**	**2,200,000**
Qdot 800	15,800,000	10,600,000	4,100,000	2,100,000
**Qdot® VIVID® 800**	**16,000,000**	**11,000,000**	**4,100,000**	**2,100,000**

A

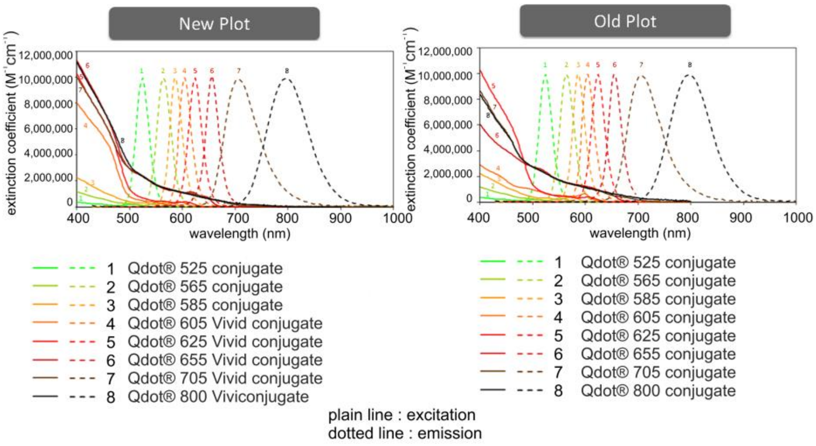

B

Information on Original and Vivid Qdots

A: Molar extinction coefficients ($M^{-1}cm^{-1}$) of original & Vivid Qdot nanocrystal streptavidin conjugates at indicated wavelengths ((4) for the original Qdots and information provided by life technology technical services for Vivid Qdots).

B : excitation and emission spectra for original and Vivid Qdots. Note that the Vivid information is not currently available in the Molecular probe book or online ; and that Qdot 625 and 800 have similar values for original and Vivid confirming that those 2 Qdots were always of the Vivid type. The currently available manual on Qdot (revised Nov-2011) secondary antibody conjugates (5) protocol does not state 585 or 605 as Vivid. Reproduced with permission from LifeTechnology
