# Supplementary material for: Working with Commercially Available Quantum Dots for Immunofluorescence on Tissue Sections
Source: PLoS One. 2016 Sep 29;11(9):e0163856. doi: 10.1371/journal.pone.0163856 (PMC5042461; doi:10.1371/journal.pone.0163856)
Supplement: S2 Fig — (PDF) [file pone.0163856.s002.pdf]

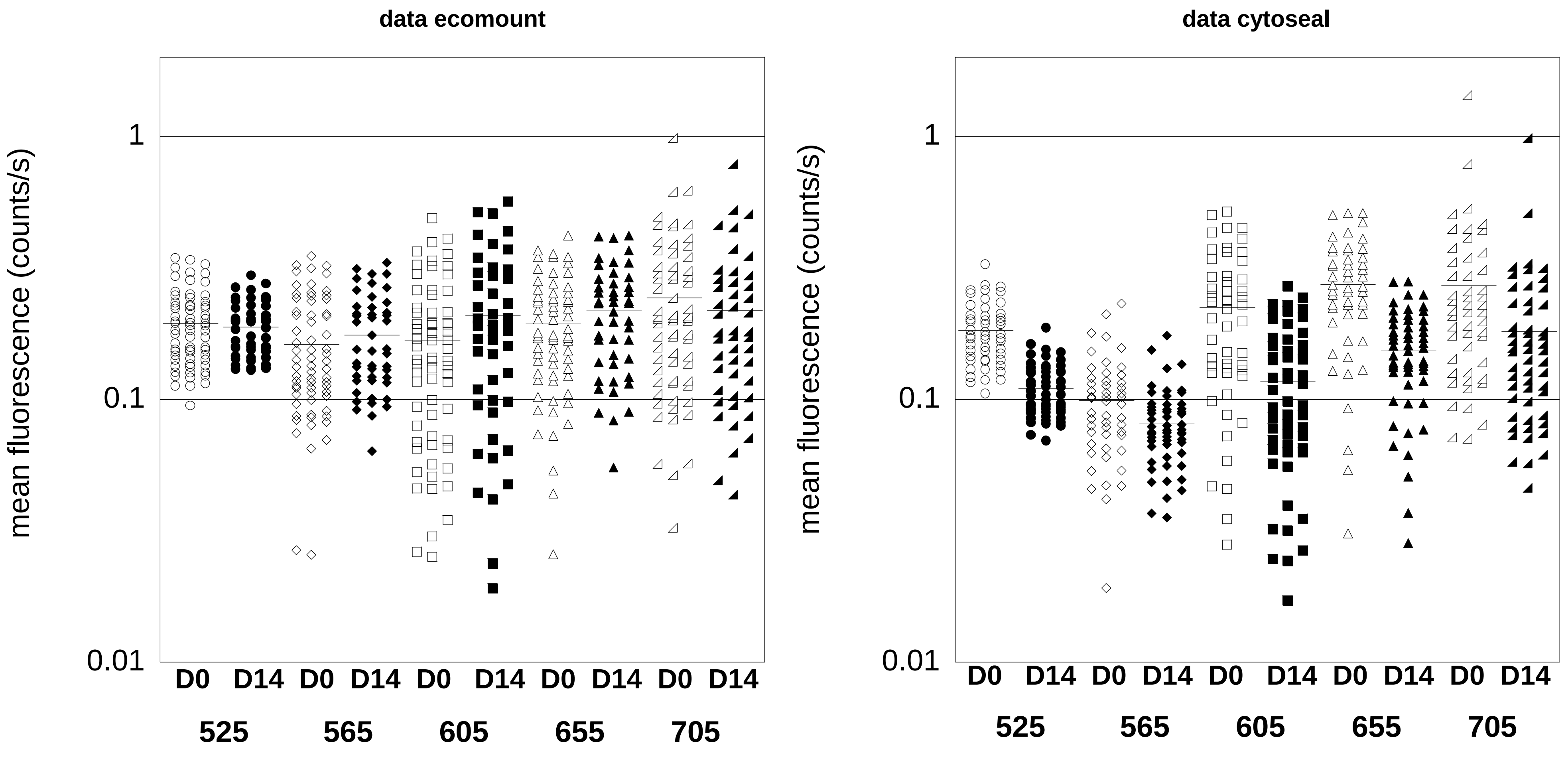

Qdots are more stable in Ecomount than in Cytoseal

The graph show the mean fluorescence intensities at day 0 and Day 14 in 2 matched fields in 2 serial sections stained at the same time with a 6 color protocol multiplex (Qdot 525, Qdot 565, Qdot 605Vivid, Qdot 655Vivid, Qdot 705 Vivid and nucear counterstain QNuclear Red) and mounted either in Ecomount (left) or Cuytoseal (right). The images were analysed using Nuance Software on unmixed intensity images for each Qdot spectrum. Each dot correspond to a region of interest thresholded for positivity of the staining. After 14 days the fluorescence of all Qdots have decreased in cytoseal but not in Ecomount (paired T test with unequal variance below).
