# Supplementary material for: Working with Commercially Available Quantum Dots for Immunofluorescence on Tissue Sections
Source: PLoS One. 2016 Sep 29;11(9):e0163856. doi: 10.1371/journal.pone.0163856 (PMC5042461; doi:10.1371/journal.pone.0163856)
Supplement: S3 Fig — (PDF) [file pone.0163856.s003.pdf]

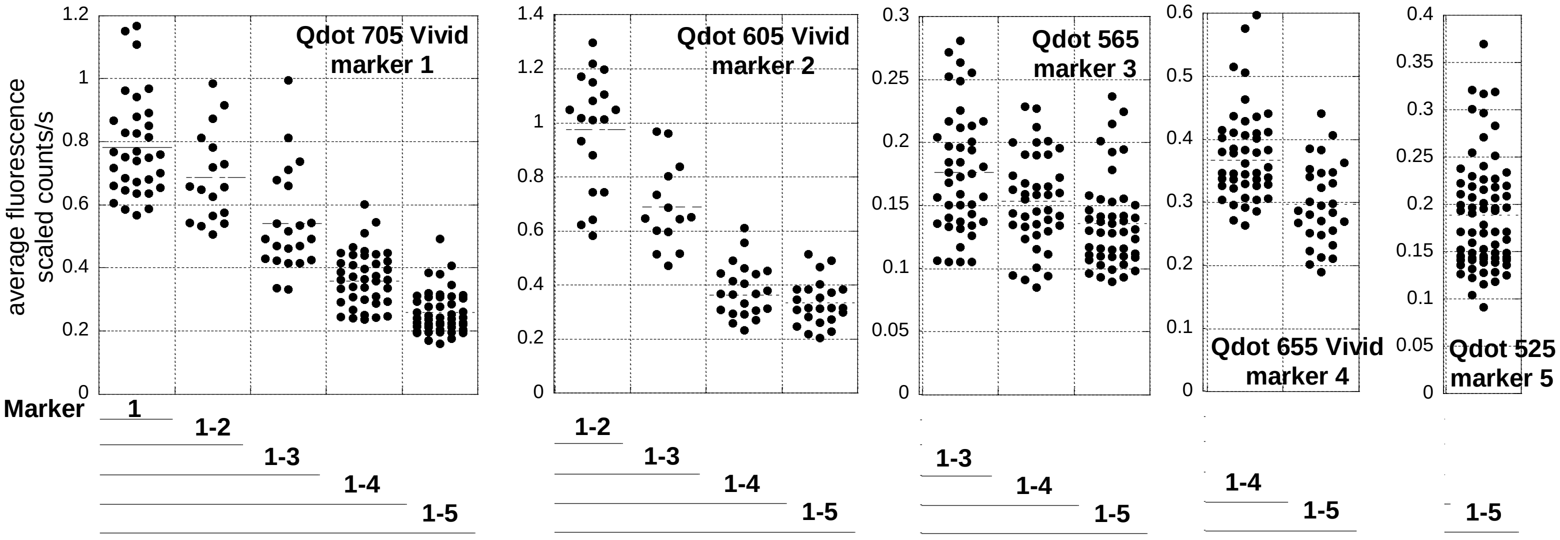

Decrease immunofluorescence during multiplex staining

Five serial sections were stained with 1 to 5 different markers : marker 1 Qdot 705Vivid, marker 2 Qdot 605 Vivid, Marker 3 Qdot 565, Marker 4 Qdot 655Vivid, Marker 5 streptavidin Qdot 525. After each staining, a slide was mounted and imaged using Nuance Fx camera. Analysis of the intensity of Qdots 705Vivid, 605Vivid, Qdot 565, Qdot 655 Vivid & Qdot 525 fluorescence are shown for one representative field of view (aligned in serial sections). Each dot corresponds to one thresholded positive cell. The intensity of fluorescence decreases with the number of staining, and this is more intense for Vivid Qdots (compare decrease of average fluorescence intensity after 3 staining for 705Vivid and 605 vivid with 565)
