# Supplementary material for: Working with Commercially Available Quantum Dots for Immunofluorescence on Tissue Sections
Source: PLoS One. 2016 Sep 29;11(9):e0163856. doi: 10.1371/journal.pone.0163856 (PMC5042461; doi:10.1371/journal.pone.0163856)
Supplement: S1 File — (PDF) [file pone.0163856.s004.pdf]

Protocol 1 : Single immunostaining with original streptavidin conjugated Qdot :

1.	Xylene	15 min
2.	Alcohol	Through 4 baths
3.	Water	2 min
4.	Retrieval EDTA (0.1mM, Ph8)	12 min @pressure
5.	Bond wash	5 min
6.	Avidin/Biotin block	20 min
7.	Primary antibodies	1 hour
8.	Bond wash	5 min
9.	Biotinylated Secondary antibody	30 min
10.	Bond wash	2x 5 min
11.	Streptavidin Qdot	1 hour
12.	Bond wash	5 min
13.	Q-nuclear red	20 min
14.	Tap water	rinse briefly
15.	Alcohol	Through 4 baths
16.	Xylene	Through 2 baths
17.	Mount in Qmount	

Leave at room temperature in the dark for 12 hours before viewing

**Item**	**Cat number**
Avidin/Biotin blocking kit	Vector SP-2001
Antibody diluent (1°Ab, Qdots)	Leica, AR9352
Background reducing diluent(2°Ab)	DAKO, S3022
Bond Wash	Leica, AR9590
Q nuclear red	LifeTechnologies Q10363
Mounting medium	InVitrogen Q10336- discontinued

Protocol 2 : 4 color immunostaining with original streptavidin conjugated Qdot :

1.	Xylene	15 min
2.	Alcohol	Through 4 baths
3.	Water	2 min
4.	Retrieval EDTA (0.1mM, Ph8)	12 min @pressure
5.	Bond wash	5 min
6.	Avidin/Biotin block (a few drops)	20 min
7.	serum-free protein block	10 min
8.	Primary antibodies 1 + 2	Overnight at 4 degres
9.	Bond wash	5 min
10.	Ist Biotinylated Secondary antibody	30 min
11.	Bond wash	2x 5 min
12.	Ist Streptavidin Qdot	30 min
13.	Avidin/Biotin block	20 min
14.	serum-free protein block	10 min
15.	2nd Biotinylated Secondary antibody	30 min
16.	Bond wash	2x5 min
17.	2nd Streptavidin Qdot	30 min
18.	Avidin/Biotin block	20 min
19.	serum-free protein block	10 min
20.	Primary antibodies 3 + 4	1 hour room temperature
21.	Bond wash	5 min
22.	3rd Biotinylated Secondary antibody	30 min
23.	Bond wash	2x5 min
24.	3rd Streptavidin Qdot	30 min

25. Avidin/Biotin block — 20 min
26. serum-free protein block — 10 min
27. 4th Biotinylated Secondary antibody — 30 min
28. Bond wash — 2x5 min
29. 4th Streptavidin Qdot — 30 min
30. Bond wash — 5 min
31. Q-nuclear red — 20 min
32. Tap water — rinse briefly
33. Alcohol — Through 4 baths
34. Xylene — Through 2 baths
35. Mount in Qmount

Item	Cat number
Avidin/Biotin blocking kit	Vector SP-2001
Antibody diluent (1°Ab, Qdots)	Leica, AR9352
Background reducing diluent(2°Ab)	DAKO, S3022
Serum-free protein block	Dako, X0909
Bond Wash	Leica, AR9590
Q nuclear red	LifeTechnologies Q10363
Mounting medium	InVitrogen Q10336- discontinued

Protocol 3 : 4 color multiplex immunostaining with Qdots conjugated secondary antibodies

1. Xylene — 15 min
2. Alcohol — Through 4 baths
3. Water — 2 min
4. Retrieval EDTA (0.1mM, Ph8) — 12 min @pressure
5. Bond wash — 5 min
6. serum-free protein block — 10 min
7. Primary antibodies 1 — Overnight at 4 degres
8. Bond wash — 3 min
9. Ist Qdot conjugated Secondary antibody — 30 min
10. Bond wash — 3 min
11. Fab 1/25 — 10 min
12. serum-free protein block — 10 min
13. Primary antibodies 2 — 1 hour
14. Bond wash — 3 min
15. 2nd Qdot conjugated Secondary antibody — 30 min
16. Bond wash — 3 min
17. Fab 1/25 — 10 min
18. serum-free protein block — 10 min
19. Primary antibodies 3 — 1 hour
20. Bond wash — 3 min
21. 3rd Qdot conjugated Secondary antibody — 30 min
22. Bond wash — 3 min
23. Fab 1/25 — 10 min
24. serum-free protein block — 10 min
25. Primary antibodies 1 — 1 hour
26. Bond wash — 3 min
27. 4th Qdot conjugated Secondary antibody — 30 min
28. Bond wash — 3 min
29. Fab 1/25 — 10 min
30. serum-free protein block — 10 min
31. Bond wash — 5 min

32. Q-nuclear red — 20 min
33. Tap water — rinse briefly
34. Alcohol — Through 4 baths
35. Xylene — Through 2 baths
36. Mount in cytoseal or Ecomount

**Item**	**Cat number**
Avidin/Biotin blocking kit	Vector SP-2001
Antibody diluent (1°Ab, Qdots)	Leica, AR9352
Background reducing diluent(2°Ab)	DAKO, S3022
Serum-free protein block	Dako, X0909
Bond Wash	Leica, AR9590
Q nuclear red	LifeTechnologies Q10363
Mounting medium	Cytoseal Richard-Allen Scientific 8310-4<br>Ecomount MP-EM897-100

Protocol 4 : Final method
Example for 4 Qdot conjugated secondary and 1 streptavidin Qdot.

1.	Xylene	15 min
2.	Alcohol	Through 4 baths
3.	Water	2 min
4.	Retrieval EDTA (0.1mM, Ph8)	12 min @pressure
5.	TBS wash	5 min
6.	Avidin/Biotin block (a few drops)	20 min
7.	BKRA/Tris wash	10 min
8.	serum-free protein block	10 min
9.	Primary antibodies 1	Overnight at 4 degrees
10.	BKRA/Tris wash	3 min
11.	Ist Qdot conjugated Secondary antibody	35 min
12.	BKRA/Tris wash	3 min
13.	Fab	2x10 min
14.	serum-free protein block	10 min
15.	Primary antibodies 2	35 min
16.	BKRA/Tris wash	3 min
17.	2nd Qdot conjugated Secondary antibody	35 min
18.	BKRA/Tris wash	3 min
19.	Fab	10 min
20.	serum-free protein block	10 min
21.	Primary antibodies 3	35 min
22.	BKRA/Tris wash	3 min
23.	3rd Qdot conjugated Secondary antibody	35 min
24.	BKRA/Tris wash	3 min
25.	Fab	10 min
26.	serum-free protein block	10 min
27.	Primary antibodies 4	35 min
28.	BKRA/Tris wash	3 min
29.	4th Qdot conjugated Secondary antibody	30 min
30.	BKRA/Tris wash	3 min
31.	Fab	10 min
32.	serum-free protein block	10 min
33.	Primary antibodies 5	35 min
34.	BKRA/Tris wash	3 min
35.	Biotinylated Secondary antibody	35 min
36.	BKRA/Tris wash	3 min
37.	Streptavidin Qdot	30 min
38.	BKRA/Tris wash	5 min
39.	Q-nuclear red	20 min
40.	BKRA/Tris wash rinse briefly	1 min
41.	Alcohol	Through 4 baths
42.	Xylene	Through 2 baths
43.	Mount in Ecomount	

Item	Cat number
Peroxidase block	S2023
Serum free protein block	X0909
Background reducing diluent (2°Ab)	S3022
A/B blocking kit	SP-2001
Ecomount	MP-EM897-100

All secondary antibodies are diluted in Background reducing diluent with 1 % of the appropriate serum
Fab diluted in Background reducing diluent with 1 % of the appropriate serum (1/25 for αRabbit 1/50 for αmouse)

The protocol can be scaled to the appropriate number of markers preliminary. Cross reactivity need to be tested prior to use in multiplex. Sera, Fab and secondaries need to be carefully chosen to prevent cross reactivity.

**Primary antibodies**		
Sigma	HPA000635	HMOX1 rabbit anti-human antibody (HO1)
Serotec	MCA1853	CD163 Mouse anti-human antibody
Sigma	HPA004114	CD206 rabbit anti-human antibody
Abcam	ab32144	Anti-CCR2 [E68] antibody (ab32144)
Abcam	Ab6671	Anti-TNF alpha antibody
Abcam	Ab7856	Anti MHCII
Dako	M087601-2	CD68, clone PG-M1
**Secondary Ab**		
Dako	E043201-8	Rabbit Ig biotinylated
Abcam	ab6899	Donkey anti-sheep IgG H+L (Biotin)
Abcam	Ab97258	Goat polyclonal Secondary Antibody to Mouse IgG3
Dako	E043301-2	Mouse Ig Biotinylated
Sigma	SAB3700976	Anti-Rabbit IgG (H+L), F(ab) fragment antibody produced in donkey
Sigma	R5506	Anti-Rabbit IgG (whole molecule) antibody produced in goat
**Qdot conjugated secondary Ab**		
Life Technologies	Q22074	Qdot 525 Donkey anti-rabbit IgG conjugate
Life Technologies	Q11631MP	Qdot 565 goat F(ab')2 anti-Rat IgG conjugate
Life Technologies	Q22079	Qdot 585 Donkey anti-mouse IgG conjugate
Life Technologies	Q22083	Qdot 605 Donkey anti-rabbit IgG conjugate
Life Technologies	Q11022MP	Qdot 655 goat F(ab')2 anti-mouse IgG conjugate
Life Technologies	Q11461MP	Qdot 705 goat F(ab')2 anti-rabbit IgG conjugate
Life Technologies	Q22073	Qdot™ 525 donkey anti-mouse IgG conjugate (H+L)
Life Technologies	Q22076	Qdot™ 565 donkey anti-mouse IgG conjugate (H+L)
Life Technologies	Q22077	Qdot™ 565 donkey anti-rabbit IgG conjugate (H+L)
Life Technologies	Q22080	Qdot™ 585 donkey anti-rabbit IgG conjugate (H+L)
Life Technologies	Q22082	Qdot™ 605 donkey anti-mouse IgG conjugate (H+L)
Life Technologies	Q22085	Qdot™ 625 donkey anti-mouse IgG conjugate (H+L)
Life Technologies	Q22086	Qdot™ 625 donkey anti-rabbit IgG conjugate (H+L)
Life Technologies	Q22088	Qdot™ 655 donkey anti-mouse IgG conjugate (H+L), Qdot™ VIVID
Life Technologies	Q22089	Qdot™ 655 donkey anti-rabbit IgG conjugate (H+L), Qdot™ VIVID
Life Technologies	Q11062MP	Qdot™ 705 Goat F(ab´)2 Anti-Mouse IgG Conjugate (H+L), 1 µM solution highly cross-adsorbed
**Qdot streptavidin conjugates**		
Invitrogen	Q10141MP	Qdot® 525 Streptavidin Conjugate
Invitrogen	Q10131MP	Qdot® 565 Streptavidin Conjugate
Invitrogen	Q10101MP	Qdot® 605 Streptavidin Conjugate
Invitrogen	A10196	Qdot® 625 Streptavidin Conjugate
Invitrogen	Q10121MP	Qdot® 655 Streptavidin Conjugate
Invitrogen	Q10161MP	Qdot® 705 Streptavidin Conjugate
Invitrogen	Q10111MP	Q dot 585

**Mounting media**		
A Menarini diagnostics	MP-EM897-100	Ecomount
Invitrogen	Q10336	Q mount Q dot mounting media
Thermo Fisher	8310-4	Richard Allan Scientific cytoseal 60
**Others**		
Dako	S3022	background reducing antibody diluent
Dako	S2023	Peroxidase-Blocking Solution, Dako REAL™
Dako	X0909	protein block, serum free
Sigma	D9663-10ml	Donkey serum
Sigma	G6767-500ml	Goat serum
Invitrogen	Q10363	Qnuclear deep red stain
Life Technologies	Q20001MP	Qdot incubation buffer
Stratech Scientific	715-007-003	AffiniPure Fab fragment Donkey Anti-Mouse IgG (H+L)
Stratech Scientific	711-007-003	AffiniPure Fab fragment Donkey Anti-Rabbit IgG (H+L)
Vector	SP-2001	Avidin/biotin blocking kit
