# Supplementary material for: Working with Commercially Available Quantum Dots for Immunofluorescence on Tissue Sections
Source: PLoS One. 2016 Sep 29;11(9):e0163856. doi: 10.1371/journal.pone.0163856 (PMC5042461; doi:10.1371/journal.pone.0163856)
Supplement: S2 File — (PDF) [file pone.0163856.s005.pdf]

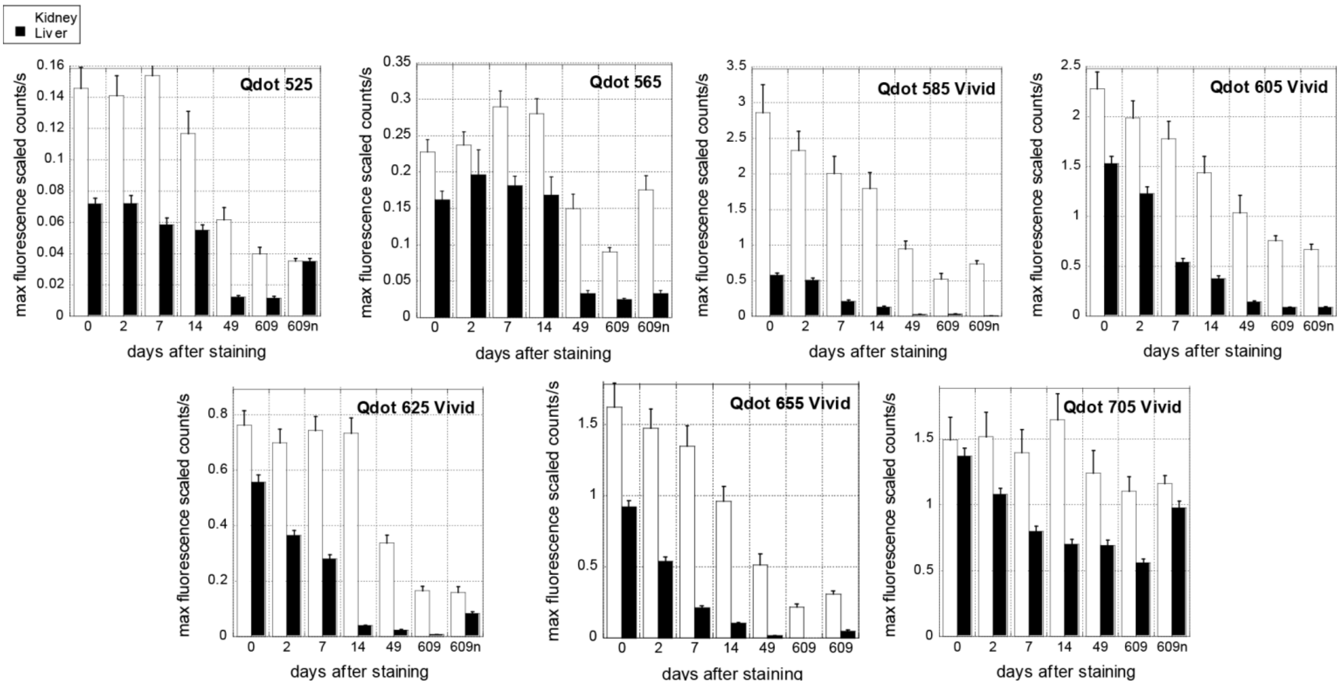

a : Max fluorescence intensity

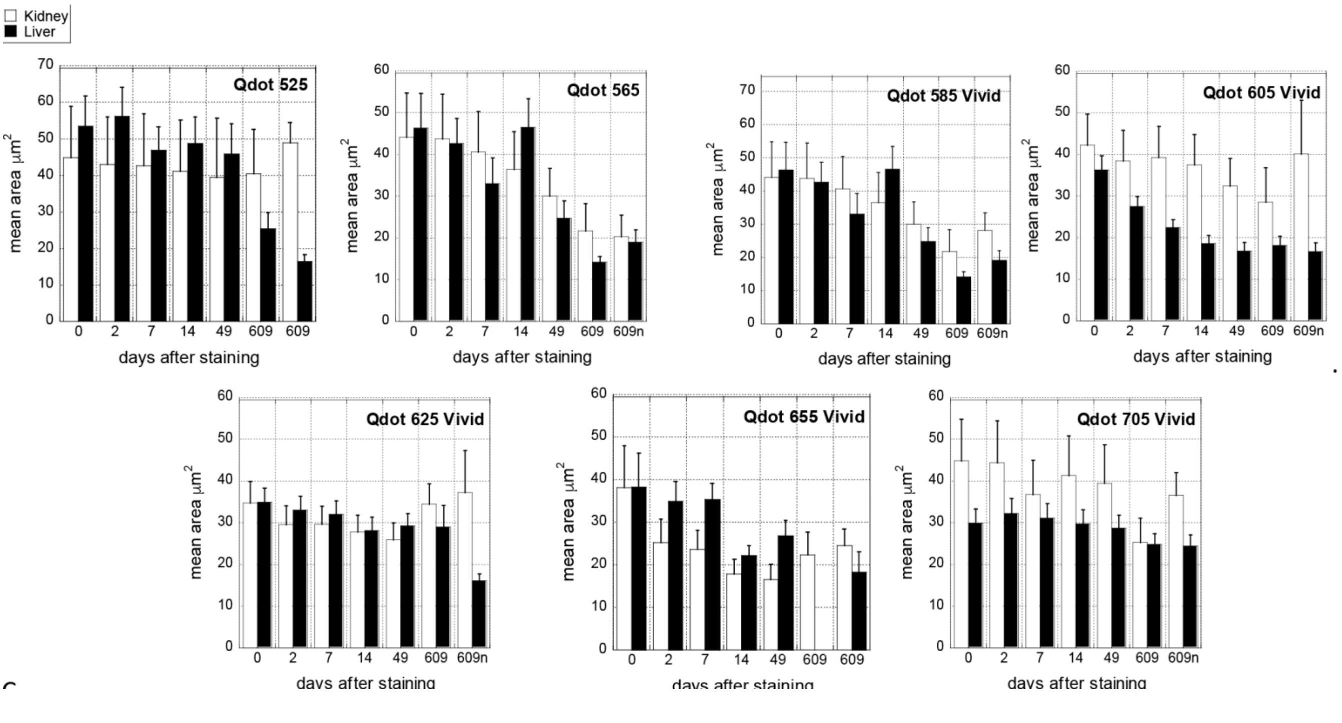

b : Mean area analysed

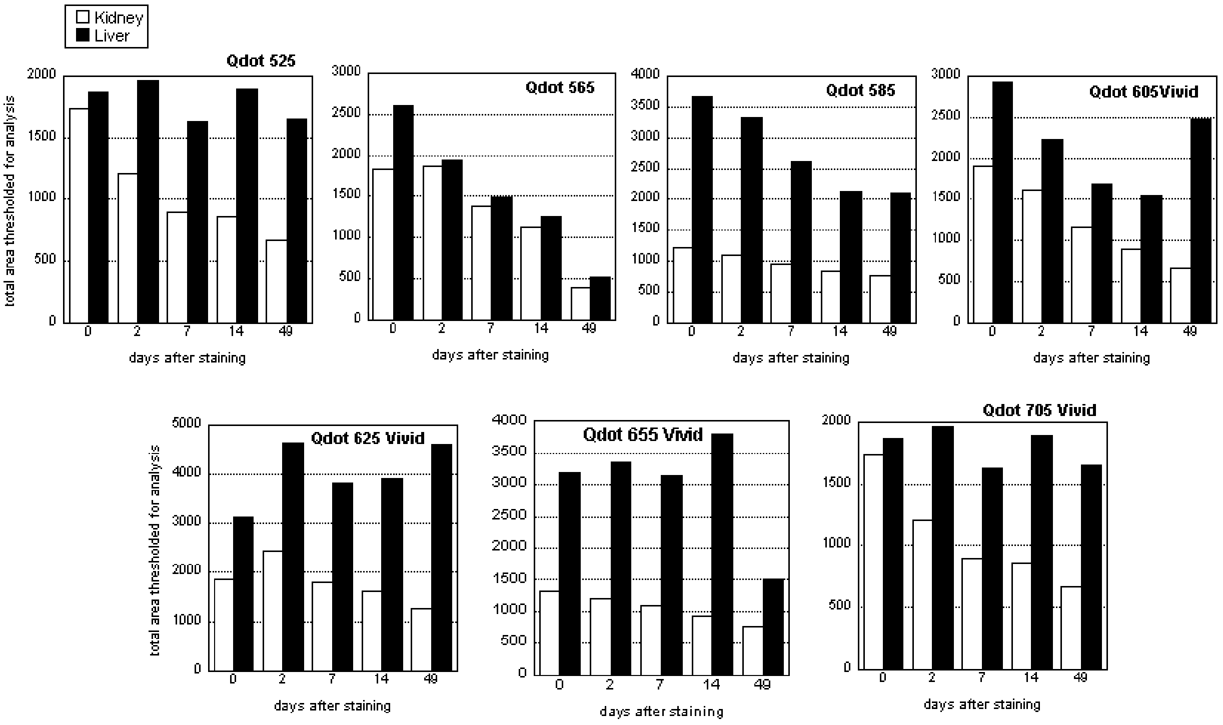

c : Total area analysed

Figure A : Testing long term fluorescence stability in Cytoseal over 20 months
a : Max fluorescence intensity
b : Mean area analysed
c: Total area analysed

Serial sections of a block containing both liver and a kidney were stained with the same primary Ab and a secondary Ab directly conjugated with the indicated Qdot. The "same" area was imaged at the indicated times after staining and intensity of fluorescence was obtained using Nuance software. The average fluorescence is shown in Fig 5.

Here are presented the data for Maximum fluorescence, Average areas and Total area analysed

A decrease in fluorescence often leads to a decrease in the area thresholded which may not be obvious in graphs representing the *mean area thresholded* as a minimum area of 100pixels is imposed.

The graphs below show the *total area thresholded*. Note that the total area thresholded in Liver sample is often more stable, despite a decrease in fluorescence intensity (eg liver 655 in Fig 5 & S2 File , while it may decrease when mean and max fluorescence seem to remain constant (eg kidney 705 in Fig 5 & S2 File Figure A a & b). This indicates that all parameters must be taken into account to judge on stability of the fluorescence

525

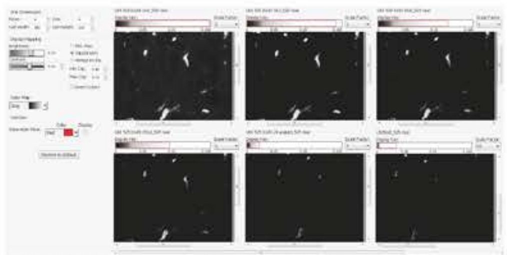

Liver Qdot 525 brightness 0.29 ; contrast 0.20
scale factors 1,1,1,1,4, 10 for 0,2,7,14 & 49 days after staining

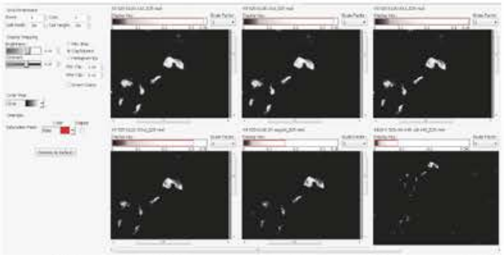

Kidney Qdot 525 brightness 0.29 ; contrast 0.20
scale factors 1,1,1,1,2,4 for 0,2,7,14 & 49 days after staining

565

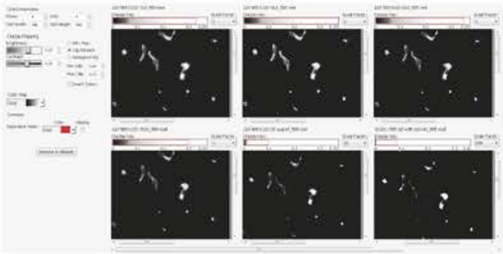

Liver Qdot 565Vivid brightness 0.29 ; contrast 0.20
scale factors 1,1,1,10,100 for 0,2,7,14 & 49 days after staining

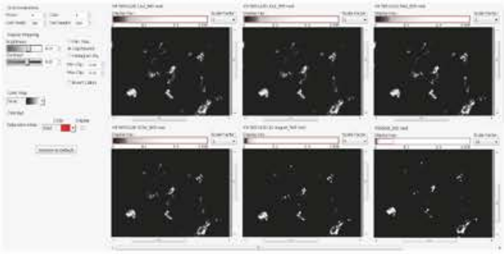

Kidney Qdot 565 brightness 0.29 ; contrast 0.20
scale factors 1,1,1,1,4, 10 for 0,2,7,14 & 49 days after staining

585

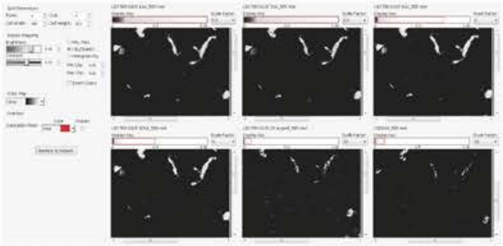

Liver Qdot 585Vivid brightness 0.48 ; contrast 0.20
scale factors 0.5,0.5,2,4,10,10 for 0,2,7,14 & 49 days after staining

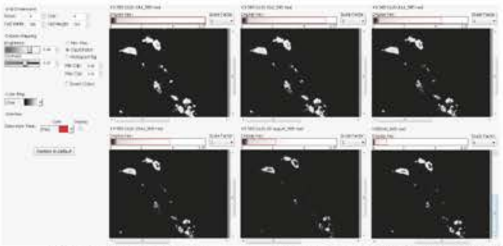

Kidney Qdot 585Vivid brightness 0.48 ; contrast 0.20
scale factors 1,1,1,1,1,4 for 0,2,7,14 & 49 days after staining

605

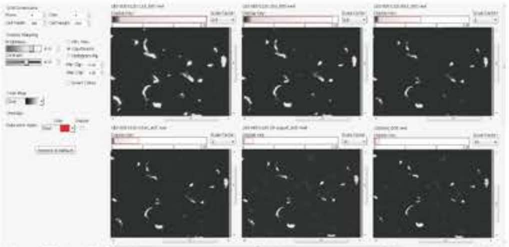

Liver Qdot 605Vivid brightness 0.53 ; contrast 0.20
scale factors 0.5,0.5,1,2,10, 10 for 0,2,7,14 & 49 days after staining

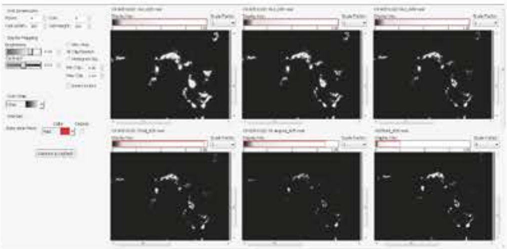

Kidney Qdot 605Vivid brightness 0.48 ; contrast 0.00
scale factors 1,1,1,1,4, for 0,2,7,14 & 49 days after staining

625

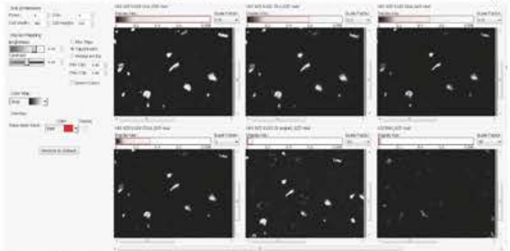

Liver Qdot 625Vivid brightness 0.48 ; contrast 0.00
scale factors 0.5,0.5,0.5,1,10,10 for 0,2,7,14 & 49 days after staining

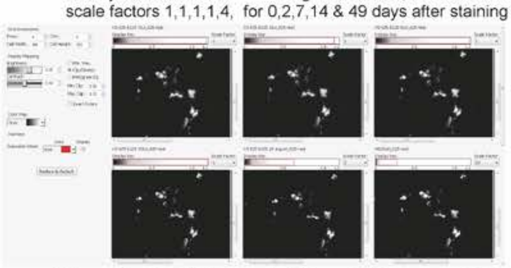

Kidney Qdot 625Vivid brightness 0.48 ; contrast 0.00
scale factors 1,1,1,1,4, for 0,2,7,14 & 49 days after staining

655

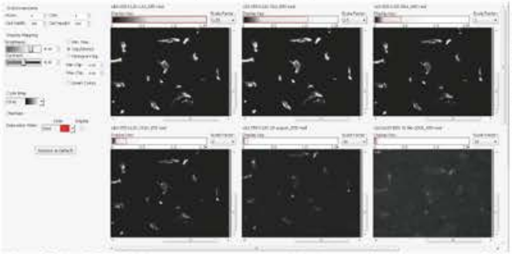

Liver Qdot 655Vivid brightness 0.48 ; contrast 0.00
scale factors 0.25,0.5,1,2,10, 10 for 0,2,7,14 & 49 days after staining

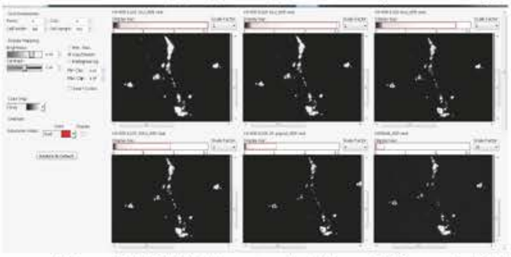

Kidney Qdot 655Vivid brightness 0.48 ; contrast 0.00
scale factors 1,1,1,2,4,10 for 0,2,7,14 & 49 days after staining

705

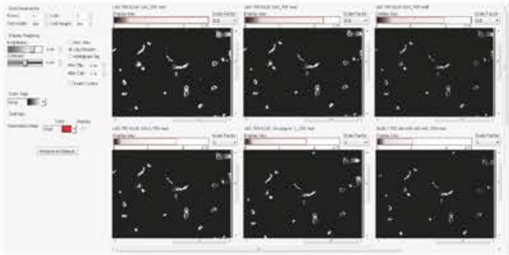

Liver Qdot 705Vivid brightness 0.48 ; contrast 0.00
scale factors 0.5,0.5,0.5,1,1 for 0,2,7,14 & 49 days after staining

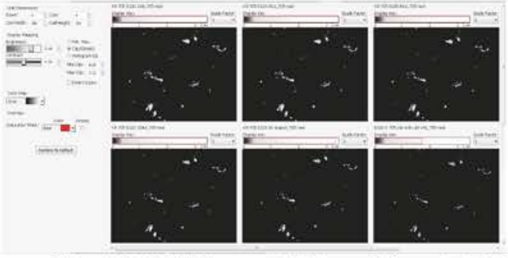

Kidney Qdot 705Vivid brightness 0.48 ; contrast 0.00
scale factors 1,1,1,1,1,1 for 0,2,7,14 & 49 days after staining

Figure B : Intensity comparison of the same fields taken at indicated days after staining (0,1,3,8,14,49). The images represent a quarter of the total field of view imaged and captured for analysis. They are “clipstretch” intensity images (scaled counts/s) obtained in the Nuance software (option which maps the lowest 0.01% of the pixels to 0, the highest 0.01% to 255, and linearly interpolates in between those values, preventing a few bright or saturated pixels from skewing the display).
The red rectangle scale above each image represent the minimum and maximum intensity value within the image . In order to be able to visualise the changes in intensity of fluorescence with time, the brightness and contrast may have been changed (this affects similarly all the images displayed) as indicated while the scales of each images may be changed as indicated . The scale factor is for visual display only and does not affect the scale. A scaling of 2 will double the intensity of the image, while a scaling of 0.5 half the intensity. Liver : left panels, Kidney : right panels

Sections stained with Qdot525
Liver: the scale factor for the first 4 images (0 to 14 days) is the same, and the shape and size of the positive cells remain similar – However the display key confirm that the intensity of fluorescence is slowly decreasing (0-0.168 at 0days 0- <0.10 after 14 days). **The stability is not appropriate for quantification**
Kidney: The fluorescence is more stable within the first 14 days (4 first images) with an intensity of fluorescence varying only slightly from 0.36 to 0.31. **The stability is appropriate for quantification**
Better in kidney than liver

Sections stained with Qdot565
Liver: No change in scale factor has been applied for the 4 first images (day 0 to 14) as the maximum fluorescence does not change with time. The staining is stable for at least 14 days and is suitable for quantification. **The stability is appropriate for quantification**
Kidney : No change in scale factor has been applied for the 4 first images (day 0 to 14) as the maximum fluorescence does not change with time. The staining is stable for at least 14 days and is suitable for quantification. **The stability is appropriate for quantification**

Sections stained with Qdot585 Vivid
Liver : fluorescence stable for 2 days, with subsequent loss of fluorescence intensity. **not appropriate for quantification**
Kidney : Although the scale factor is the same and the shape and intensity of the cells seem similar there is a steady decrease in the intensity of fluorescence. Fluorescence fairly stable for 2 days. **The stability is not appropriate for quantification**

Sections stained with Qdot605 Vivid
Liver : steady decrease of fluorescence intensity over time . **not appropriate for quantification**
Kidney : although the display scale remains the same for the first 14 days (0-3.85) showing that the maximum fluorescence (which is due here to a small number of very bright pixels that are not decreasing in intensity with time) does not change , there is a steady loss of fluorescence . **The stability is not appropriate for quantification**

Sections stained with Qdot625 Vivid
Liver : fluorescence decreases steadily with time. **not appropriate for quantification**
Kidney : fluorescence stable for at least 14 days **- The stability is appropriate for quantification**
Better in kidney than liver

Sections stained with Qdot655 Vivid
Liver : fluorescence decreases steadily with time. **not appropriate for quantification**
Kidney : fluorescence stable for 2 days, with subsequent loss of fluorescence intensity. **not appropriate for quantification.**
Better in kidney than liver

Sections stained with Qdot705 Vivid
Liver : fluorescence decreases steadily with time. **not appropriate for quantification**
Kidney : fluorescence stable for at least 14 days **The stability is appropriate for quantification**
Better in kidney than liver
